# Crystal structures and conformational analyses of three pyranochromene derivatives

**DOI:** 10.1107/S2056989015012967

**Published:** 2015-07-15

**Authors:** K. Swaminathan, K. Sethusankar, G. Siva Kumar, M. Bakthadoss

**Affiliations:** a Department of Physics, RKM Vivekananda College (Autonomous), Chennai 600 004, India; b Department of Organic Chemistry, University of Madras, Guindy Campus, Chennai 600 025, India

**Keywords:** crystal structure, pyran­ochromene, coumarin derivatives, mol­ecular sheets, inversion dimers, chains, hydrogen bonding

## Abstract

The title compounds, C_27_H_20_O_6_, (I) [systematic name: methyl 7-oxo-14-phenyl-1*H*,7*H*,14*H*-pyrano[3,2-*c*:5,4-*c*′]dichromene-14a(6b*H*)-carboxyl­ate], C_24_H_22_O_5_, (II) [systematic name: methyl 1-oxo-6-phenyl-2,3,4,12b-tetra­hydro-1*H*,6*H*-chromeno[3,4-*c*]chromene-6a(7*H*)-carboxyl­ate], and C_25_H_23_N_3_O_4_, (III) [systematic name: 6-(4-ethyl­phen­yl)-2,4-dimethyl-1,3-dioxo-2,3,4,12b-tetra­hydro-1*H*,6*H*-chromeno[4′,3′:4,5]pyrano[2,3-*d*]pyrimidine-6a(7*H*)-carbo­nitrile], are pyran­ochromene derivatives. The central pyran rings (*B*) of compounds (I) and (III) adopt half-chair conformations, whereas that of compound (II) adopts a sofa conformation. The pyran rings (*A*) of the chromene ring systems of compounds (II) and (III) adopt half-chair conformations, while that of compound (I) adopts a sofa conformation. The mean plane of the central pyran rings (*B*) make dihedral angles of 70.02 (6), 61.52 (6) and 69.12 (7)°, respectively, with the mean planes of the chromene moieties (*C*+*A*) of compounds (I), (II) and (III). The bicyclic coumarin ring system (*C*+*A*+*B*+*E*) in compound (I) is almost planar (r.m.s. deviation = 0.042 Å). The carbo­nitrile side chain in compound (III) is very nearly linear, with the C—C≡N angle being 176.6 (2)°. The cyclo­hexene ring (*E*), fused with the central pyran ring (*B*) in compound (II) adopts a sofa conformation. In the mol­ecular structures of compounds (II) and (III), there are C—H⋯O short contacts, which generate *S*(7) ring motifs. In the crystal structures of the title compounds, mol­ecules are linked by C—H⋯O hydrogen bonds, which generate mol­ecular sheets parallel to the *ab* plane, with *R*_4_^3^(28) loops in (I), inversion dimers with *R*_2_^2^(10) loops in (II) and chains along [010] with *R*_2_^2^(12) ring motifs in (III). In the crystal structures of (I) and (III), there are also C—H⋯π inter­actions present, leading to the formation of a three-dimensional framework in (II) and to sheets parallel to (101) in (III).

## Abstract 2

In the crystal structures of the three title pyran–chromene derivatives, (I)–(III), mol­ecules are linked by C—H⋯O hydrogen bonds which generate mol­ecular sheets parallel to the *ab* plane with 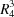
(28) loops in (I), inversion dimers with 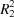
(10) loops in (II) and chains along the *b* axis with 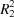
(12) ring motifs in (III).

## Chemical context   

Chromenes, the oxygen-containing heterocyclic scaffolds, represent a privileged structural motif, well distributed in biologically active natural products and also in synthetic compounds used in the fields of medicine, agrochemistry, cosmetics and pigments. A number of drugs containing chromene are used in the treatment of ailments such as hypertension, asthma, ischemia and urinary incontinence. Chromene derivatives are known to possess anti­tumor, anti­vascular (Gourdeau *et al.*, 2004 ▸), anti­microbial (Sangani *et al.*, 2012 ▸), anti-oxidant (Mladenović *et al.*, 2011 ▸), anti­fungal (Thareja *et al.*, 2010 ▸), anti­viral (Smith *et al.*, 1998 ▸), anti-inflammatory (Moon *et al.*, 2007 ▸), anti­malarial (de Andrade-Neto *et al.*, 2004 ▸), sex hormonal (Mohr *et al.*, 1975 ▸), anti­proliferative (Bianchi & Tava, 1987 ▸), anti­cancer, anti-Alzheimer, anti-Parkinson and Huntington’s diseases (Andrani & Lapi, 1960 ▸; Zhang *et al.*, 1982 ▸), Tumor Necrosis Factor (TNF–α) inhibitory (Cheng *et al.*, 2003 ▸), estrogenic (Jain *et al.*, 2009 ▸), anti­filaricidal (Tripathi *et al.*, 2000 ▸) and anti­convulsant (Bhat *et al.*, 2008 ▸) activities.
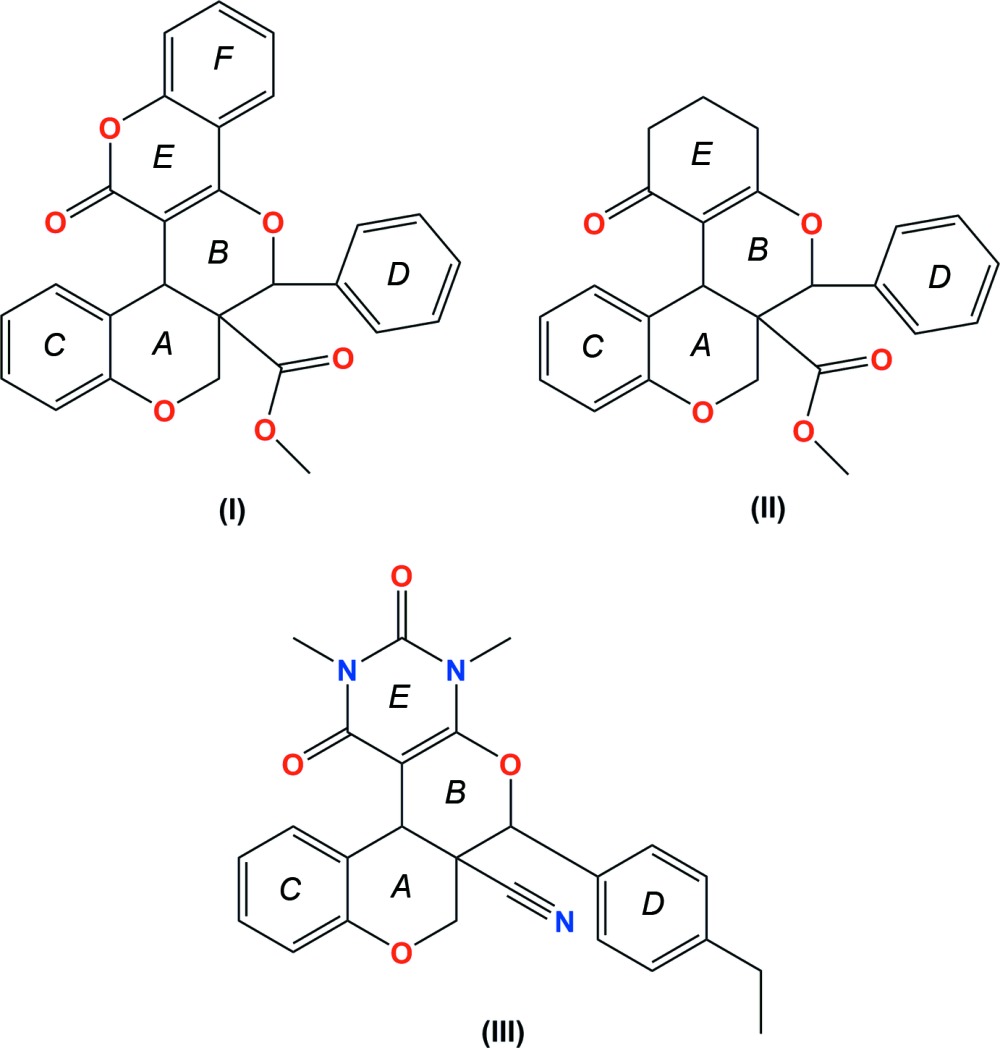


Chromene derivatives also play an important role in the production of highly effective fluorescent dyes for synthetic fibers, daylight-fluorescent pigments and electrophotographic and electroluminescent devices (Khairy *et al.*, 2009 ▸). Against this background, the title compounds, (I), (II) and (III), were synthesized and we report herein on their crystal structures and mol­ecular conformations.

## Structural commentary   

**Figure 1:**
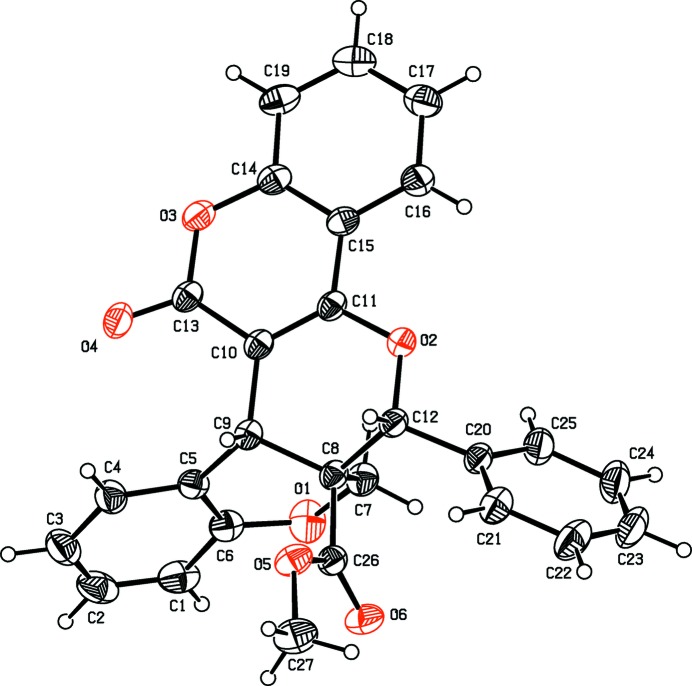
The mol­ecular structure of compound (I), showing the atom labelling. Displacement ellipsoids are drawn at the 30% probability level.

The mol­ecular structures of compounds, (I), (II) and (III) are illustrated in Figs. 1 ▸, 2 ▸ and 3 ▸, respectively. All three compounds comprise a central pyran ring (*B*) fused with a chromene ring system (*C*+*A*). The central pyran ring (*B*) is fused with a second chromene ring system (*E*+*F*) in (I), a cyclo­hexene ring (*E*) in (II) and a pyrimidine ring (*E*) in (III); see scheme and Figs. 1 ▸–3 ▸ ▸. In compounds (I) and (II), a carboxyl­ate side chain and a benzene ring (*D*) are attached to the central pyran ring (*B*), in adjacent positions, whereas in (III) there is a cabo­nitrile side chain and an ethyl-substituted benzene ring attached to the central pyran ring (*B*).

In compounds (I) and (III), the central pyran rings (*B*) adopt half-chair conformations with puckering amplitudes *Q* = 0.5166 (15) Å, θ = 51.22 (17), φ = 259.4 (2)° and *Q* = 0.486 (2) Å, θ = 128.3 (2), φ = 74.5 (3)°, respectively. In compound (II), the central pyran ring (*B*) adopts a sofa conformation [*Q* = 0.5614 (15) Å, θ = 58.41 (15), φ = 286.21 (16)°]. The cyclo­hexene ring (*E*) fused to the central pyran ring (*B*) in compound (II), adopts a sofa conformation [*Q* = 0.497 (2) Å, θ = 109.8 (2), φ = 5.9 (2)°]. The pyran ring (*A*) of the chromene moiety adopts a half-chair conformation in compounds (II) and (III) [*Q* = 0.4850 (14) Å, θ = 53.26 (17), φ = 271.70 (19)° and *Q* = 0.507 (2) Å, θ = 128.9 (2), φ = 92.7 (3)°, respectively] and a sofa conformation in compound (I) [*Q* = 0.5130 (16) Å, θ = 57.83 (18), φ = 234.6 (2)°].

In compound (I), the dihedral angle between the benzene ring (C) and the mean plane of the pyran ring (*A* – sofa conformation) of the chromene moiety is 14.95 (8)°, whereas in (II) and (III) the same angles are 7.83 (7) and 6.42 (10)°, respectively (the *A* rings here have half-chair conformations). The decrease in the value of the dihedral angle in compounds (II) and (III) is probably due to the intra­molecular C—H⋯O short contacts which generate *S*(7) ring motifs. The second coumarin ring system (*E*+*F*) is almost planar with the dihedral angle between the pyran and benzene rings being 3.73 (7)°. Atom O4 deviates from the mean plane of this coumarin ring system by 0.111 (1) Å. The phenyl ring (*D*) is inclined to the mean plane of the central pyran ring (*B*), by 60.48 (8)°.

In compound (II), the mean plane of the central pyran ring (*B*) makes dihedral angles of 22.63 (8) and 56.99 (9)° with the mean plane of the six-membered carbocylic ring (*E*) and the phenyl ring (*D*), respectively. Atom O3 deviates from the mean plane of ring (*E*) by 0.199 (1) Å.

In compound (III), the central pyran ring (*B*) makes dihedral angles of 7.36 (9) and 58.24 (10)° with the pyrimidine (*E*) and ethyl-substituted benzene (*D*) rings, respectively. Atom O3 and the methyl group C atom, C16, deviate significantly from the mean plane of the pyrimidine ring (*E*) by 0.106 (1) and −0.107 (2) Å, respectively.

## Supra­molecular features   

**Figure 4:**
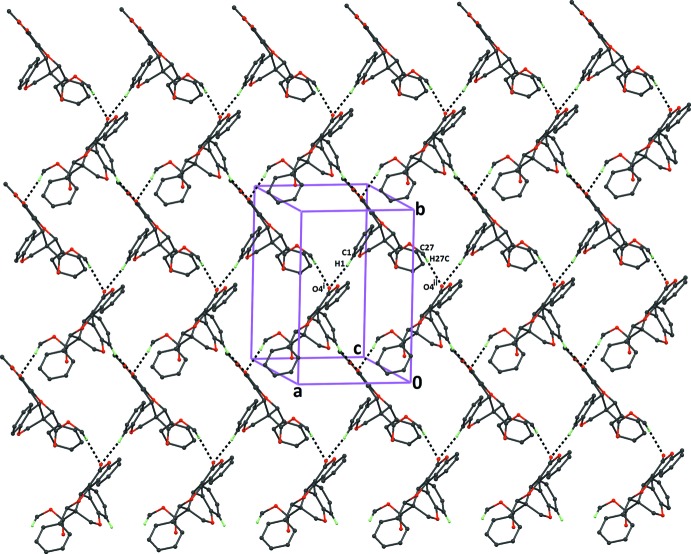
The crystal packing of compound (I), viewed along the *c* axis, showing the formation of two-dimensional mol­ecular sheets running parallel to the *ab* plane. Dashed lines indicate the inter­molecular C—H⋯O inter­actions (Table 1 ▸). H atoms not involved in hydrogen bonding have been excluded for clarity.

In compound (I), C—H⋯O hydrogen bonds are present in which the carboxyl­ate and chromene ring C atoms, C27 and C1, respectively, act as donors and the coumarin ring O atom, O4, acts as a single acceptor (Table 1 ▸). These hydrogen bonds link the mol­ecules into 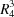
(28) ring motifs, resulting in the formation of sheets parallel to the *ab* plane (Fig. 4 ▸). The sheets are linked by C—H⋯π inter­actions, forming a three-dimensional framework (Table 1 ▸).

**Figure 5:**
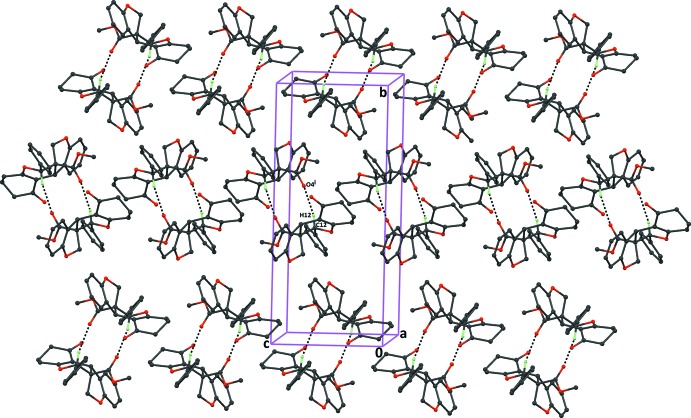
The crystal packing of the title compound (II), viewed along the *a* axis, showing the formation of inversion dimers with the descriptor 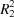
(10). Dashed lines indicate the inter­molecular C—H⋯O inter­actions (Table 2 ▸). H atoms not involved in hydrogen bonding have been excluded for clarity.

In compound (II), mol­ecules are linked through pairs of C–H⋯O hydrogen bonds, resulting in the formation of inversion dimers with graph-set motif 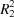
(10) (Table 2 ▸ and Fig. 5 ▸).

**Figure 6:**
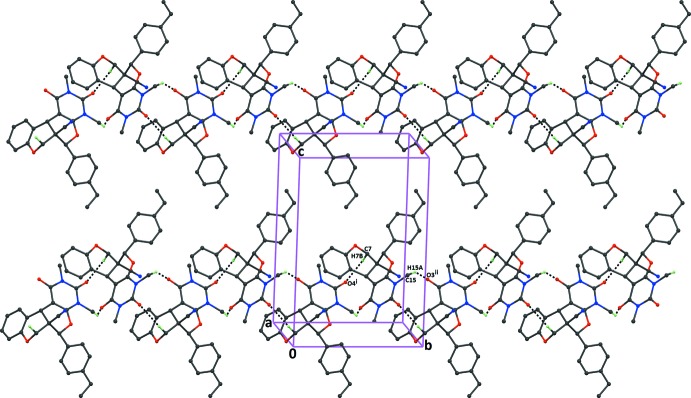
The crystal packing of the title compound (III), viewed along the *a* axis, showing the formation of adjacent 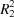
(12) ring motifs which connect the inversion-related mol­ecules into chains along [010]. Dashed lines indicate the inter­molecular C—H⋯O inter­actions (Table 3 ▸). H atoms not involved in hydrogen bonding have been excluded for clarity.

In compound (III), mol­ecules are linked through C—H⋯O hydrogen bonds, resulting in the formation chains along the *b*-axis direction, enclosing 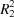
(12) ring motifs (Fig. 6 ▸). The chains are linked by C—H⋯π inter­actions, forming sheets parallel to (101) (Table 3 ▸).

## Database survey   

A search of the Cambridge Structural Database (Version 5.36, last update February 2015; Groom & Allen, 2014 ▸) for 4,4a,5,10b-tetra­hydro-4-phenyl­pyrano[3,4-*c*]chromene yielded 14 hits. The bond distances and bond angles in compounds (I)–(III) are in agreement with those in the reported structures. For example: compounds (I) and (II) exhibits structural similarities with entries LESWIR (Ponnusamy *et al.*, 2013 ▸) which has a toluene rather than a phenyl substituent on ring (*B*), OLEZIP (Kathiravan & Raghunathan, 2010 ▸) which has a 4-meth­oxy­phenyl substituent, and AZUKIQ (Swaminathan *et al.*, 2011 ▸) which has a 2-chloro­phenyl substituent. Compound (III) is similar to entries WUNNAV (Bakthadoss *et al.*, 2009 ▸), AXACAE (Kanchanadevi *et al.*, 2011 ▸) and WUNNEZ (Bakthadoss *et al.*, 2009 ▸), but only the last compound also has a cabo­nitrile side chain.

## Synthesis and crystallization   

**Compound (I)**: A mixture of (*E*)-methyl 2-[(2-formyl­phen­oxy)meth­yl]-3-phenyl­acrylate (0.296 g, 1 mmol) and 4-hy­droxy-2*H* -chromen-2-one (0.162 g, 1 mmol) was placed in a round bottom flask and heated at 453 K for 1 h. After completion of the reaction as indicated by TLC, the crude product was washed with 5 ml of ethyl­acetate and hexane mixture (1:49 ratio) which successfully provided compound (I) as a colourless solid. Single crystals suitable for X-ray diffraction were prepared by slow evaporation of a solution of (I) in ethyl­acetate at room temperature.

**Compound (II)**: A mixture of (*E*)-methyl 2-[(2-formyl­phen­oxy)meth­yl]-3-phenyl­acrylate (0.296 g, 1 mmol) and cyclo­hexane-1,3-dione (0.112 g, 1 mmol) was placed in a round bottom flask and heated at 453 K for 1 h. After completion of the reaction as indicated by TLC, the crude product was washed with 5 ml of ethyl­acetate and hexane mixture (1:49 ratio) which successfully provided the crude product of compound (II) as a colourless solid. Single crystals suitable for X-ray diffraction were prepared by slow evaporation of a solution of (II) in ethyl­acetate at room temperature.

**Compound (III)**: A mixture of (*E*)-2-[(2-formyl­phen­oxy)meth­yl]-3-(4-ethyl­phen­yl)acrylo­nitrile (0.291 g, 1 mmol) and 1,3-di­methyl­pyrimidine-2,4,6(1*H*,3*H*,5*H*)-trione (0.156 g, 1 mmol) was placed in a round-bottom flask and heated at 453 K for 1 h. After completion of the reaction as indicated by TLC, the crude product was washed with 5 ml of ethyl­acetate and hexane mixture (1:49 ratio) which successfully provided pure compound (III) as a colourless solid. Single crystals suitable for X-ray diffraction were prepared by slow evaporation of a solution of (III) in ethyl­acetate at room temperature.

## Refinement   

Crystal data, data collection and structure refinement details for compounds (I), (II) and (III) are summarized in Table 4 ▸. The positions of all of the H atoms were located in difference electron density maps. During refinement they were treated as riding atoms, with *d*(C—H) = 0.93, 0.96, 0.97 and 0.98 Å for aryl, methyl, methyl­ene and methine H atoms, respectively, and with *U*_iso_(H)= 1.5*U*_eq_(C) for methyl H atoms and 1.2*U*_eq_(C) for other H atoms.

## Supplementary Material

Crystal structure: contains datablock(s) I, II, III, global. DOI: 10.1107/S2056989015012967/su5160sup1.cif

Structure factors: contains datablock(s) I. DOI: 10.1107/S2056989015012967/su5160Isup2.hkl

Structure factors: contains datablock(s) II. DOI: 10.1107/S2056989015012967/su5160IIsup3.hkl

Structure factors: contains datablock(s) III. DOI: 10.1107/S2056989015012967/su5160IIIsup4.hkl

Supporting information file. DOI: 10.1107/S2056989015012967/su5160Isup5.cml

Supporting information file. DOI: 10.1107/S2056989015012967/su5160IIsup6.cml

Supporting information file. DOI: 10.1107/S2056989015012967/su5160IIIsup7.cml

CCDC references: 1410607, 1410606, 1410605

Additional supporting information:  crystallographic information; 3D view; checkCIF report

## Figures and Tables

**Figure 2:**
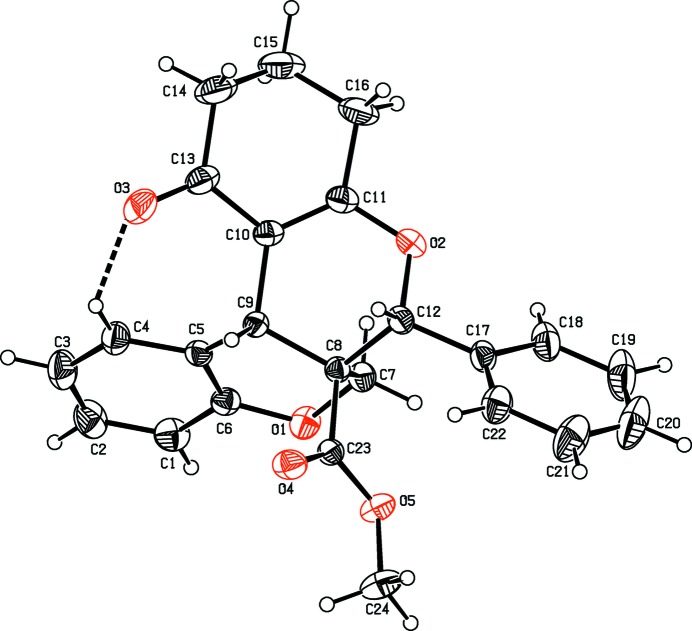
The mol­ecular structure of compound (II), with the atom labelling. The intra­molecular C4—H4⋯O3 inter­action, which generates an *S*(7) ring motif, is shown as a dashed line. Displacement ellipsoids are drawn at the 30% probability level.

**Figure 3:**
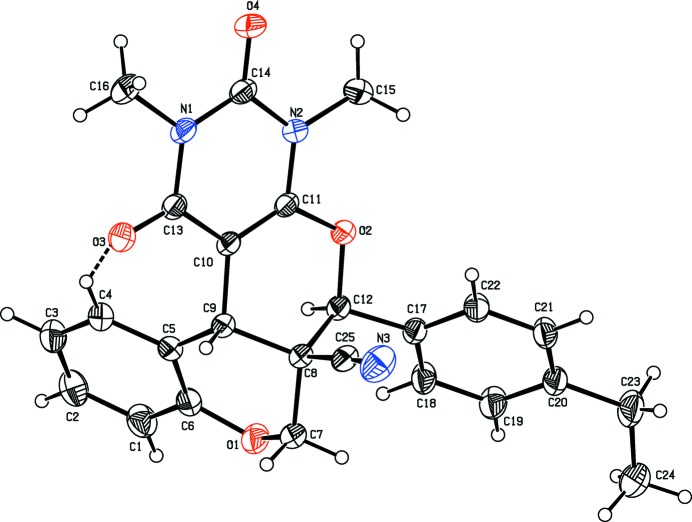
The mol­ecular structure of compound (III), with the atom labelling. The intra­molecular C4—H4⋯O3 inter­action, which generates an *S*(7) ring motif, is shown as a dashed line. Displacement ellipsoids are drawn at the 30% probability level.

**Table 1:** Hydrogen-bond geometry (Å, °) for (I) *Cg*1 and *Cg*2 are the centroids of rings C14–C19 and C1–C6, respectively.

*D*—H⋯*A*	*D*—H	H⋯*A*	*D*⋯*A*	*D*—H⋯*A*
C1—H1⋯O4^i^	0.93	2.58	3.411 (2)	149
C27—H27*C*⋯O4^ii^	0.96	2.37	3.053 (2)	128
C12—H12⋯*Cg*1^iii^	0.98	2.73	3.6861 (17)	166
C18—H18⋯*Cg*2^iv^	0.93	2.84	3.674 (2)	150

Symmetry codes: (i) 
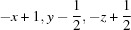
; (ii) 
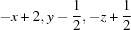
; (iii) 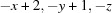; (iv) 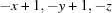.

**Table 2:** Hydrogen-bond geometry (Å, °) for (II)

*D*—H⋯*A*	*D*—H	H⋯*A*	*D*⋯*A*	*D*—H⋯*A*
C4—H4⋯O3	0.93	2.22	2.973 (2)	138
C12—H12⋯O4^i^	0.98	2.41	3.3613 (18)	164

Symmetry code: (i) 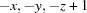.

**Table 3:** Hydrogen-bond geometry (Å, °) for (III) *Cg*1 and *Cg*2 are the centroids of rings C14–C19 and C1–C6, respectively.

*D*—H⋯*A*	*D*—H	H⋯*A*	*D*⋯*A*	*D*—H⋯*A*
C4—H4⋯O3	0.93	2.39	3.155 (3)	139
C7—H7*B*⋯O4^i^	0.97	2.52	3.423 (3)	156
C15—H15*A*⋯O3^ii^	0.96	2.50	3.315 (3)	143
C16—H16*C*⋯*Cg*1^iii^	0.96	2.93	3.739 (2)	143
C24—H24*A*⋯*Cg*2^iv^	0.96	2.70	3.634 (3)	164

Symmetry codes: (i) 
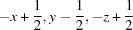
; (ii) 
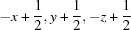
; (iii) 
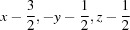
; (iv) 
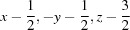
.

**Table 4:** Experimental details

	(I)	(II)	(III)
Crystal data			
Chemical formula	C_27_H_20_O_6_	C_24_H_22_O_5_	C_25_H_23_N_3_O_4_
*M*_r_	440.43	390.42	429.46
Crystal system, space group	Monoclinic, *P*2_1_/*c*	Monoclinic, *P*2_1_/*c*	Monoclinic, *P*2_1_/*n*
Temperature (K)	296	296	296
*a*, *b*, *c* (Å)	9.3980 (15), 14.0050 (12), 15.9890 (13)	11.1694 (10), 20.1405 (19), 8.5835 (7)	11.4471 (5), 11.2076 (4), 16.5407 (7)
β (°)	92.048 (5)	96.453 (3)	91.990 (2)
*V* (Å^3^)	2103.1 (4)	1918.7 (3)	2120.80 (15)
*Z*	4	4	4
Radiation type	Mo *K*α	Mo *K*α	Mo *K*α
μ (mm^−1^)	0.10	0.09	0.09
Crystal size (mm)	0.35 × 0.30 × 0.25	0.35 × 0.30 × 0.25	0.35 × 0.30 × 0.25

Data collection			
Diffractometer	Bruker Kappa APEXII CCD	Bruker Kappa APEXII CCD	Bruker Kappa APEXII CCD
Absorption correction	Multi-scan (*SADABS*; Bruker, 2008 ▸)	Multi-scan (*SADABS*; Bruker, 2008 ▸)	Multi-scan (*SADABS*; Bruker, 2008 ▸)
*T*_min_, *T*_max_	0.966, 0.976	0.968, 0.977	0.968, 0.977
No. of measured, independent and observed [*I* > 2σ(*I*)] reflections	19091, 3698, 2964	23342, 5466, 3694	18281, 3715, 2814
*R*_int_	0.027	0.033	0.030
(sin θ/λ)_max_ (Å^−1^)	0.595	0.699	0.595

Refinement			
*R*[*F* ^2^ > 2σ(*F* ^2^)], *wR*(*F* ^2^), *S*	0.037, 0.106, 1.07	0.047, 0.155, 0.99	0.044, 0.126, 1.03
No. of reflections	3698	5466	3715
No. of parameters	299	263	292
H-atom treatment	H-atom parameters constrained	H-atom parameters constrained	H-atom parameters constrained
Δρ_max_, Δρ_min_ (e Å^−3^)	0.15, −0.20	0.26, −0.22	0.33, −0.28

Computer programs: *APEX2* and *SAINT* (Bruker, 2008 ▸), *SHELXS97* and *SHELXL97* (Sheldrick, 2008 ▸), *ORTEP-3 for Windows* (Farrugia, 2012 ▸), *Mercury* (Macrae *et al.*, 2008 ▸) and *PLATON* (Spek, 2009 ▸).

## supplementary crystallographic information

### (I) Methyl 7-oxo-14-phenyl-1H,7H,14H-pyrano[3,2-c:5,4-c']dichromene-14a(6bH)-carboxylate] Crystal data

**Table:**

C_27_H_20_O_6_	*F*(000) = 920
*M**_r_* = 440.43	*D*_x_ = 1.391 Mg m^−^^3^
Monoclinic, *P*2_1_/*c*	Mo *K*α radiation, λ = 0.71073 Å
Hall symbol: -P 2ybc	Cell parameters from 3698 reflections
*a* = 9.3980 (15) Å	θ = 2.6–25.0°
*b* = 14.0050 (12) Å	µ = 0.10 mm^−^^1^
*c* = 15.9890 (13) Å	*T* = 296 K
β = 92.048 (5)°	Block, colourless
*V* = 2103.1 (4) Å^3^	0.35 × 0.30 × 0.25 mm
*Z* = 4	

### (I) Methyl 7-oxo-14-phenyl-1H,7H,14H-pyrano[3,2-c:5,4-c']dichromene-14a(6bH)-carboxylate] Data collection

**Table:**

Bruker Kappa APEXII CCD diffractometer	3698 independent reflections
Radiation source: fine-focus sealed tube	2964 reflections with *I* > 2σ(*I*)
Graphite monochromator	*R*_int_ = 0.027
ω & φ scans	θ_max_ = 25.0°, θ_min_ = 2.6°
Absorption correction: multi-scan (SADABS; Bruker, 2008)	*h* = −11→11
*T*_min_ = 0.966, *T*_max_ = 0.976	*k* = −16→16
19091 measured reflections	*l* = −19→19

### (I) Methyl 7-oxo-14-phenyl-1H,7H,14H-pyrano[3,2-c:5,4-c']dichromene-14a(6bH)-carboxylate] Refinement

**Table:**

Refinement on *F*^2^	Primary atom site location: structure-invariant direct methods
Least-squares matrix: full	Secondary atom site location: difference Fourier map
*R*[*F*^2^ > 2σ(*F*^2^)] = 0.037	Hydrogen site location: inferred from neighbouring sites
*wR*(*F*^2^) = 0.106	H-atom parameters constrained
*S* = 1.07	*w* = 1/[σ^2^(*F*_o_^2^) + (0.0556*P*)^2^ + 0.3545*P*] where *P* = (*F*_o_^2^ + 2*F*_c_^2^)/3
3698 reflections	(Δ/σ)_max_ < 0.001
299 parameters	Δρ_max_ = 0.15 e Å^−^^3^
0 restraints	Δρ_min_ = −0.20 e Å^−^^3^

### (I) Methyl 7-oxo-14-phenyl-1H,7H,14H-pyrano[3,2-c:5,4-c']dichromene-14a(6bH)-carboxylate] Special details

**Table:**

Geometry. All e.s.d.'s (except the e.s.d. in the dihedral angle between two l.s. planes) are estimated using the full covariance matrix. The cell e.s.d.'s are taken into account individually in the estimation of e.s.d.'s in distances, angles and torsion angles; correlations between e.s.d.'s in cell parameters are only used when they are defined by crystal symmetry. An approximate (isotropic) treatment of cell e.s.d.'s is used for estimating e.s.d.'s involving l.s. planes.
Refinement. Refinement of *F*^2^ against ALL reflections. The weighted *R*-factor *wR* and goodness of fit *S* are based on *F*^2^, conventional *R*-factors *R* are based on *F*, with *F* set to zero for negative *F*^2^. The threshold expression of *F*^2^ > σ(*F*^2^) is used only for calculating *R*-factors(gt) *etc*. and is not relevant to the choice of reflections for refinement. *R*-factors based on *F*^2^ are statistically about twice as large as those based on *F*, and *R*- factors based on ALL data will be even larger.

### (I) Methyl 7-oxo-14-phenyl-1H,7H,14H-pyrano[3,2-c:5,4-c']dichromene-14a(6bH)-carboxylate] Fractional atomic coordinates and isotropic or equivalent isotropic displacement parameters (Å^2^)

**Table:**

	*x*	*y*	*z*	*U*_iso_*/*U*_eq_	
C1	0.56782 (17)	0.20825 (13)	0.27702 (12)	0.0602 (5)	
H1	0.5188	0.1513	0.2680	0.072*	
C2	0.55034 (19)	0.25923 (16)	0.34916 (12)	0.0673 (5)	
H2	0.4893	0.2366	0.3892	0.081*	
C3	0.6227 (2)	0.34377 (16)	0.36281 (11)	0.0641 (5)	
H3	0.6102	0.3782	0.4117	0.077*	
C4	0.71382 (17)	0.37705 (13)	0.30365 (10)	0.0527 (4)	
H4	0.7636	0.4335	0.3136	0.063*	
C5	0.73273 (15)	0.32771 (11)	0.22924 (9)	0.0418 (3)	
C6	0.65935 (16)	0.24239 (11)	0.21752 (10)	0.0472 (4)	
C7	0.77878 (16)	0.20819 (11)	0.09054 (10)	0.0473 (4)	
H7A	0.7343	0.2434	0.0444	0.057*	
H7B	0.8165	0.1494	0.0682	0.057*	
C8	0.90161 (15)	0.26711 (10)	0.12772 (9)	0.0382 (3)	
C9	0.84184 (14)	0.35891 (10)	0.16676 (9)	0.0373 (3)	
H9	0.9199	0.3912	0.1977	0.045*	
C10	0.79072 (15)	0.42388 (10)	0.09597 (9)	0.0377 (3)	
C11	0.83533 (15)	0.41354 (10)	0.01665 (9)	0.0390 (3)	
C12	1.00608 (15)	0.29584 (10)	0.05912 (9)	0.0398 (3)	
H12	1.0776	0.3390	0.0844	0.048*	
C13	0.69743 (15)	0.50314 (10)	0.11408 (10)	0.0406 (3)	
C14	0.68064 (15)	0.54090 (10)	−0.03296 (10)	0.0436 (4)	
C15	0.78006 (15)	0.47146 (10)	−0.05170 (9)	0.0416 (3)	
C16	0.81551 (18)	0.45965 (12)	−0.13507 (10)	0.0519 (4)	
H16	0.8829	0.4143	−0.1490	0.062*	
C17	0.7513 (2)	0.51477 (13)	−0.19664 (11)	0.0594 (5)	
H17	0.7757	0.5067	−0.2521	0.071*	
C18	0.65055 (19)	0.58214 (13)	−0.17657 (12)	0.0592 (5)	
H18	0.6063	0.6183	−0.2188	0.071*	
C19	0.61517 (17)	0.59612 (12)	−0.09496 (11)	0.0534 (4)	
H19	0.5482	0.6420	−0.0815	0.064*	
C20	1.08306 (16)	0.21352 (10)	0.01989 (9)	0.0437 (4)	
C21	1.21663 (18)	0.18833 (12)	0.05144 (12)	0.0557 (4)	
H21	1.2581	0.2227	0.0957	0.067*	
C22	1.2892 (2)	0.11241 (13)	0.01770 (14)	0.0712 (6)	
H22	1.3792	0.0961	0.0393	0.085*	
C23	1.2289 (2)	0.06125 (13)	−0.04743 (15)	0.0754 (6)	
H23	1.2784	0.0108	−0.0706	0.091*	
C24	1.0960 (2)	0.08450 (14)	−0.07837 (13)	0.0715 (6)	
H24	1.0545	0.0487	−0.1217	0.086*	
C25	1.0226 (2)	0.16088 (12)	−0.04583 (11)	0.0572 (4)	
H25	0.9329	0.1769	−0.0680	0.069*	
C26	0.98572 (15)	0.20813 (11)	0.19238 (9)	0.0423 (4)	
C27	1.1726 (2)	0.21330 (15)	0.29418 (13)	0.0713 (5)	
H27A	1.1191	0.1816	0.3360	0.107*	
H27B	1.2367	0.2584	0.3206	0.107*	
H27C	1.2261	0.1670	0.2641	0.107*	
O1	0.67314 (12)	0.18517 (9)	0.14893 (8)	0.0638 (3)	
O2	0.93013 (11)	0.34696 (7)	−0.00690 (6)	0.0466 (3)	
O3	0.64390 (11)	0.55763 (7)	0.04799 (7)	0.0490 (3)	
O4	0.66214 (12)	0.52699 (8)	0.18248 (7)	0.0533 (3)	
O5	1.07607 (11)	0.26285 (8)	0.23656 (7)	0.0535 (3)	
O6	0.97557 (13)	0.12343 (8)	0.20042 (8)	0.0613 (3)	

### (I) Methyl 7-oxo-14-phenyl-1H,7H,14H-pyrano[3,2-c:5,4-c']dichromene-14a(6bH)-carboxylate] Atomic displacement parameters (Å^2^)

**Table:**

	*U*^11^	*U*^22^	*U*^33^	*U*^12^	*U*^13^	*U*^23^
C1	0.0410 (9)	0.0613 (11)	0.0790 (13)	0.0020 (8)	0.0107 (8)	0.0229 (10)
C2	0.0509 (10)	0.0891 (15)	0.0631 (11)	0.0121 (10)	0.0165 (9)	0.0304 (11)
C3	0.0585 (11)	0.0886 (14)	0.0459 (9)	0.0167 (10)	0.0109 (8)	0.0091 (9)
C4	0.0509 (9)	0.0614 (10)	0.0459 (9)	0.0092 (8)	0.0045 (7)	0.0014 (8)
C5	0.0351 (7)	0.0456 (8)	0.0450 (8)	0.0042 (6)	0.0036 (6)	0.0054 (7)
C6	0.0362 (8)	0.0487 (9)	0.0570 (9)	0.0018 (7)	0.0052 (7)	0.0070 (8)
C7	0.0492 (9)	0.0389 (8)	0.0540 (9)	−0.0077 (7)	0.0055 (7)	−0.0035 (7)
C8	0.0379 (8)	0.0322 (7)	0.0448 (8)	−0.0014 (6)	0.0049 (6)	−0.0016 (6)
C9	0.0339 (7)	0.0345 (7)	0.0435 (8)	−0.0020 (6)	0.0033 (6)	−0.0032 (6)
C10	0.0347 (7)	0.0305 (7)	0.0480 (8)	−0.0041 (6)	0.0031 (6)	−0.0022 (6)
C11	0.0383 (8)	0.0297 (7)	0.0492 (8)	−0.0024 (6)	0.0043 (6)	−0.0021 (6)
C12	0.0419 (8)	0.0325 (7)	0.0452 (8)	0.0012 (6)	0.0054 (6)	0.0000 (6)
C13	0.0369 (8)	0.0310 (7)	0.0539 (9)	−0.0045 (6)	0.0043 (7)	−0.0021 (7)
C14	0.0391 (8)	0.0353 (8)	0.0565 (9)	−0.0069 (6)	0.0026 (7)	0.0040 (7)
C15	0.0423 (8)	0.0328 (7)	0.0496 (8)	−0.0055 (6)	0.0004 (6)	0.0025 (6)
C16	0.0613 (10)	0.0441 (9)	0.0504 (9)	−0.0021 (8)	0.0028 (8)	0.0013 (7)
C17	0.0722 (12)	0.0556 (11)	0.0502 (10)	−0.0088 (9)	−0.0026 (8)	0.0078 (8)
C18	0.0573 (11)	0.0555 (10)	0.0640 (11)	−0.0090 (8)	−0.0109 (9)	0.0172 (9)
C19	0.0427 (9)	0.0417 (9)	0.0754 (12)	−0.0030 (7)	−0.0057 (8)	0.0122 (8)
C20	0.0482 (9)	0.0331 (8)	0.0506 (9)	0.0006 (6)	0.0143 (7)	−0.0004 (6)
C21	0.0492 (9)	0.0460 (9)	0.0726 (11)	0.0017 (7)	0.0099 (8)	−0.0053 (8)
C22	0.0588 (11)	0.0527 (11)	0.1033 (16)	0.0151 (9)	0.0186 (11)	−0.0025 (11)
C23	0.0884 (15)	0.0408 (10)	0.0995 (15)	0.0114 (10)	0.0372 (13)	−0.0086 (10)
C24	0.0917 (15)	0.0526 (11)	0.0717 (12)	−0.0006 (10)	0.0222 (11)	−0.0203 (9)
C25	0.0646 (11)	0.0512 (10)	0.0562 (10)	0.0034 (8)	0.0090 (8)	−0.0096 (8)
C26	0.0421 (8)	0.0382 (8)	0.0473 (8)	0.0019 (6)	0.0103 (7)	0.0024 (7)
C27	0.0585 (11)	0.0751 (13)	0.0790 (13)	0.0166 (10)	−0.0165 (10)	0.0123 (10)
O1	0.0535 (7)	0.0599 (7)	0.0789 (8)	−0.0232 (6)	0.0172 (6)	−0.0120 (6)
O2	0.0547 (6)	0.0411 (6)	0.0446 (6)	0.0104 (5)	0.0106 (5)	0.0027 (5)
O3	0.0476 (6)	0.0384 (6)	0.0613 (7)	0.0071 (5)	0.0075 (5)	0.0049 (5)
O4	0.0592 (7)	0.0416 (6)	0.0596 (7)	0.0046 (5)	0.0112 (5)	−0.0091 (5)
O5	0.0465 (6)	0.0464 (6)	0.0667 (7)	0.0061 (5)	−0.0100 (5)	0.0041 (5)
O6	0.0759 (8)	0.0385 (7)	0.0699 (8)	0.0000 (6)	0.0059 (6)	0.0120 (6)

### (I) Methyl 7-oxo-14-phenyl-1H,7H,14H-pyrano[3,2-c:5,4-c']dichromene-14a(6bH)-carboxylate] Geometric parameters (Å, º)

**Table:**

C1—C2	1.372 (3)	C13—O3	1.3835 (18)
C1—C6	1.390 (2)	C14—O3	1.3715 (18)
C1—H1	0.9300	C14—C19	1.384 (2)
C2—C3	1.379 (3)	C14—C15	1.389 (2)
C2—H2	0.9300	C15—C16	1.395 (2)
C3—C4	1.379 (2)	C16—C17	1.374 (2)
C3—H3	0.9300	C16—H16	0.9300
C4—C5	1.393 (2)	C17—C18	1.382 (3)
C4—H4	0.9300	C17—H17	0.9300
C5—C6	1.389 (2)	C18—C19	1.372 (3)
C5—C9	1.5211 (19)	C18—H18	0.9300
C6—O1	1.368 (2)	C19—H19	0.9300
C7—O1	1.4239 (19)	C20—C21	1.382 (2)
C7—C8	1.522 (2)	C20—C25	1.388 (2)
C7—H7A	0.9700	C21—C22	1.383 (2)
C7—H7B	0.9700	C21—H21	0.9300
C8—C26	1.522 (2)	C22—C23	1.370 (3)
C8—C9	1.5438 (19)	C22—H22	0.9300
C8—C12	1.5512 (19)	C23—C24	1.366 (3)
C9—C10	1.517 (2)	C23—H23	0.9300
C9—H9	0.9800	C24—C25	1.385 (2)
C10—C11	1.358 (2)	C24—H24	0.9300
C10—C13	1.450 (2)	C25—H25	0.9300
C11—O2	1.3524 (17)	C26—O6	1.1973 (18)
C11—C15	1.443 (2)	C26—O5	1.3283 (18)
C12—O2	1.4430 (17)	C27—O5	1.4470 (19)
C12—C20	1.5094 (19)	C27—H27A	0.9600
C12—H12	0.9800	C27—H27B	0.9600
C13—O4	1.2015 (17)	C27—H27C	0.9600
			
C2—C1—C6	119.48 (18)	O3—C13—C10	118.41 (13)
C2—C1—H1	120.3	O3—C14—C19	117.38 (14)
C6—C1—H1	120.3	O3—C14—C15	121.15 (13)
C1—C2—C3	120.46 (16)	C19—C14—C15	121.47 (15)
C1—C2—H2	119.8	C14—C15—C16	118.34 (14)
C3—C2—H2	119.8	C14—C15—C11	117.20 (14)
C2—C3—C4	119.76 (18)	C16—C15—C11	124.41 (14)
C2—C3—H3	120.1	C17—C16—C15	120.28 (16)
C4—C3—H3	120.1	C17—C16—H16	119.9
C3—C4—C5	121.29 (18)	C15—C16—H16	119.9
C3—C4—H4	119.4	C16—C17—C18	120.28 (17)
C5—C4—H4	119.4	C16—C17—H17	119.9
C6—C5—C4	117.66 (14)	C18—C17—H17	119.9
C6—C5—C9	120.11 (13)	C19—C18—C17	120.62 (16)
C4—C5—C9	121.88 (14)	C19—C18—H18	119.7
O1—C6—C5	123.45 (13)	C17—C18—H18	119.7
O1—C6—C1	115.20 (15)	C18—C19—C14	119.00 (16)
C5—C6—C1	121.34 (16)	C18—C19—H19	120.5
O1—C7—C8	113.79 (13)	C14—C19—H19	120.5
O1—C7—H7A	108.8	C21—C20—C25	118.86 (14)
C8—C7—H7A	108.8	C21—C20—C12	119.07 (14)
O1—C7—H7B	108.8	C25—C20—C12	122.05 (14)
C8—C7—H7B	108.8	C20—C21—C22	120.54 (18)
H7A—C7—H7B	107.7	C20—C21—H21	119.7
C7—C8—C26	109.83 (12)	C22—C21—H21	119.7
C7—C8—C9	109.12 (12)	C23—C22—C21	120.14 (19)
C26—C8—C9	111.40 (11)	C23—C22—H22	119.9
C7—C8—C12	110.78 (12)	C21—C22—H22	119.9
C26—C8—C12	107.13 (11)	C24—C23—C22	119.91 (17)
C9—C8—C12	108.57 (11)	C24—C23—H23	120.0
C10—C9—C5	117.35 (11)	C22—C23—H23	120.0
C10—C9—C8	107.94 (11)	C23—C24—C25	120.64 (19)
C5—C9—C8	106.85 (11)	C23—C24—H24	119.7
C10—C9—H9	108.1	C25—C24—H24	119.7
C5—C9—H9	108.1	C24—C25—C20	119.90 (18)
C8—C9—H9	108.1	C24—C25—H25	120.0
C11—C10—C13	118.51 (13)	C20—C25—H25	120.0
C11—C10—C9	122.20 (12)	O6—C26—O5	124.50 (15)
C13—C10—C9	119.20 (12)	O6—C26—C8	124.68 (14)
O2—C11—C10	124.21 (13)	O5—C26—C8	110.77 (12)
O2—C11—C15	113.56 (12)	O5—C27—H27A	109.5
C10—C11—C15	122.19 (13)	O5—C27—H27B	109.5
O2—C12—C20	107.87 (11)	H27A—C27—H27B	109.5
O2—C12—C8	109.69 (11)	O5—C27—H27C	109.5
C20—C12—C8	114.87 (11)	H27A—C27—H27C	109.5
O2—C12—H12	108.1	H27B—C27—H27C	109.5
C20—C12—H12	108.1	C6—O1—C7	118.76 (12)
C8—C12—H12	108.1	C11—O2—C12	116.87 (11)
O4—C13—O3	115.93 (13)	C14—O3—C13	121.98 (11)
O4—C13—C10	125.67 (14)	C26—O5—C27	115.86 (13)
			
C6—C1—C2—C3	0.0 (3)	O3—C14—C15—C11	4.2 (2)
C1—C2—C3—C4	−0.3 (3)	C19—C14—C15—C11	−176.08 (13)
C2—C3—C4—C5	1.1 (3)	O2—C11—C15—C14	179.24 (12)
C3—C4—C5—C6	−1.6 (2)	C10—C11—C15—C14	1.6 (2)
C3—C4—C5—C9	−174.77 (15)	O2—C11—C15—C16	1.9 (2)
C4—C5—C6—O1	−177.19 (14)	C10—C11—C15—C16	−175.73 (14)
C9—C5—C6—O1	−3.9 (2)	C14—C15—C16—C17	−1.0 (2)
C4—C5—C6—C1	1.4 (2)	C11—C15—C16—C17	176.29 (14)
C9—C5—C6—C1	174.64 (14)	C15—C16—C17—C18	−0.3 (3)
C2—C1—C6—O1	178.06 (15)	C16—C17—C18—C19	1.2 (3)
C2—C1—C6—C5	−0.6 (2)	C17—C18—C19—C14	−0.8 (2)
O1—C7—C8—C26	66.18 (16)	O3—C14—C19—C18	179.25 (14)
O1—C7—C8—C9	−56.20 (16)	C15—C14—C19—C18	−0.5 (2)
O1—C7—C8—C12	−175.67 (12)	O2—C12—C20—C21	142.93 (14)
C6—C5—C9—C10	93.17 (16)	C8—C12—C20—C21	−94.41 (17)
C4—C5—C9—C10	−93.83 (17)	O2—C12—C20—C25	−38.48 (18)
C6—C5—C9—C8	−28.10 (18)	C8—C12—C20—C25	84.17 (18)
C4—C5—C9—C8	144.90 (14)	C25—C20—C21—C22	0.5 (2)
C7—C8—C9—C10	−71.67 (14)	C12—C20—C21—C22	179.09 (15)
C26—C8—C9—C10	166.90 (11)	C20—C21—C22—C23	−0.1 (3)
C12—C8—C9—C10	49.16 (14)	C21—C22—C23—C24	−0.9 (3)
C7—C8—C9—C5	55.38 (15)	C22—C23—C24—C25	1.5 (3)
C26—C8—C9—C5	−66.05 (14)	C23—C24—C25—C20	−1.2 (3)
C12—C8—C9—C5	176.21 (11)	C21—C20—C25—C24	0.2 (2)
C5—C9—C10—C11	−140.03 (14)	C12—C20—C25—C24	−178.41 (15)
C8—C9—C10—C11	−19.34 (17)	C7—C8—C26—O6	14.1 (2)
C5—C9—C10—C13	43.55 (18)	C9—C8—C26—O6	135.12 (15)
C8—C9—C10—C13	164.25 (12)	C12—C8—C26—O6	−106.27 (16)
C13—C10—C11—O2	175.09 (12)	C7—C8—C26—O5	−168.35 (12)
C9—C10—C11—O2	−1.4 (2)	C9—C8—C26—O5	−47.33 (15)
C13—C10—C11—C15	−7.5 (2)	C12—C8—C26—O5	71.28 (14)
C9—C10—C11—C15	176.06 (12)	C5—C6—O1—C7	7.0 (2)
C7—C8—C12—O2	56.60 (15)	C1—C6—O1—C7	−171.66 (14)
C26—C8—C12—O2	176.37 (11)	C8—C7—O1—C6	24.1 (2)
C9—C8—C12—O2	−63.21 (14)	C10—C11—O2—C12	−11.38 (19)
C7—C8—C12—C20	−65.08 (16)	C15—C11—O2—C12	171.01 (11)
C26—C8—C12—C20	54.69 (16)	C20—C12—O2—C11	169.19 (11)
C9—C8—C12—C20	175.12 (12)	C8—C12—O2—C11	43.41 (15)
C11—C10—C13—O4	−171.57 (14)	C19—C14—O3—C13	176.57 (12)
C9—C10—C13—O4	5.0 (2)	C15—C14—O3—C13	−3.7 (2)
C11—C10—C13—O3	7.88 (19)	O4—C13—O3—C14	177.07 (13)
C9—C10—C13—O3	−175.57 (11)	C10—C13—O3—C14	−2.43 (19)
O3—C14—C15—C16	−178.36 (13)	O6—C26—O5—C27	3.7 (2)
C19—C14—C15—C16	1.4 (2)	C8—C26—O5—C27	−173.81 (13)

### (I) Methyl 7-oxo-14-phenyl-1H,7H,14H-pyrano[3,2-c:5,4-c']dichromene-14a(6bH)-carboxylate] Hydrogen-bond geometry (Å, º)

Cg1 and Cg2 are the centroids of rings C14–C19 and C1–C6, respectively.

**Table:**

*D*—H···*A*	*D*—H	H···*A*	*D*···*A*	*D*—H···*A*
C1—H1···O4^i^	0.93	2.58	3.411 (2)	149
C27—H27*C*···O4^ii^	0.96	2.37	3.053 (2)	128
C12—H12···*Cg*1^iii^	0.98	2.73	3.6861 (17)	166
C18—H18···*Cg*2^iv^	0.93	2.84	3.674 (2)	150

Symmetry codes: (i) −*x*+1, *y*−1/2, −*z*+1/2; (ii) −*x*+2, *y*−1/2, −*z*+1/2; (iii) −*x*+2, −*y*+1, −*z*; (iv) −*x*+1, −*y*+1, −*z*.

### (II) Methyl 1-oxo-6-phenyl-2,3,4,12b-tetrahydro-1H,6H-chromeno[3,4-c]chromene-6a(7H)-carboxylate] Crystal data

**Table:**

C_24_H_22_O_5_	*F*(000) = 824
*M**_r_* = 390.42	*D*_x_ = 1.352 Mg m^−^^3^
Monoclinic, *P*2_1_/*c*	Mo *K*α radiation, λ = 0.71073 Å
Hall symbol: -P 2ybc	Cell parameters from 5466 reflections
*a* = 11.1694 (10) Å	θ = 2.1–29.8°
*b* = 20.1405 (19) Å	µ = 0.09 mm^−^^1^
*c* = 8.5835 (7) Å	*T* = 296 K
β = 96.453 (3)°	Block, colourless
*V* = 1918.7 (3) Å^3^	0.35 × 0.30 × 0.25 mm
*Z* = 4	

### (II) Methyl 1-oxo-6-phenyl-2,3,4,12b-tetrahydro-1H,6H-chromeno[3,4-c]chromene-6a(7H)-carboxylate] Data collection

**Table:**

Bruker Kappa APEXII CCD diffractometer	5466 independent reflections
Radiation source: fine-focus sealed tube	3694 reflections with *I* > 2σ(*I*)
Graphite monochromator	*R*_int_ = 0.033
ω & φ scans	θ_max_ = 29.8°, θ_min_ = 2.1°
Absorption correction: multi-scan (SADABS; Bruker, 2008)	*h* = −15→15
*T*_min_ = 0.968, *T*_max_ = 0.977	*k* = −28→27
23342 measured reflections	*l* = −10→11

### (II) Methyl 1-oxo-6-phenyl-2,3,4,12b-tetrahydro-1H,6H-chromeno[3,4-c]chromene-6a(7H)-carboxylate] Refinement

**Table:**

Refinement on *F*^2^	Primary atom site location: structure-invariant direct methods
Least-squares matrix: full	Secondary atom site location: difference Fourier map
*R*[*F*^2^ > 2σ(*F*^2^)] = 0.047	Hydrogen site location: inferred from neighbouring sites
*wR*(*F*^2^) = 0.155	H-atom parameters constrained
*S* = 0.99	*w* = 1/[σ^2^(*F*_o_^2^) + (0.0894*P*)^2^ + 0.1805*P*] where *P* = (*F*_o_^2^ + 2*F*_c_^2^)/3
5466 reflections	(Δ/σ)_max_ < 0.001
263 parameters	Δρ_max_ = 0.26 e Å^−^^3^
0 restraints	Δρ_min_ = −0.22 e Å^−^^3^

### (II) Methyl 1-oxo-6-phenyl-2,3,4,12b-tetrahydro-1H,6H-chromeno[3,4-c]chromene-6a(7H)-carboxylate] Special details

**Table:**

Geometry. All e.s.d.'s (except the e.s.d. in the dihedral angle between two l.s. planes) are estimated using the full covariance matrix. The cell e.s.d.'s are taken into account individually in the estimation of e.s.d.'s in distances, angles and torsion angles; correlations between e.s.d.'s in cell parameters are only used when they are defined by crystal symmetry. An approximate (isotropic) treatment of cell e.s.d.'s is used for estimating e.s.d.'s involving l.s. planes.
Refinement. Refinement of *F*^2^ against ALL reflections. The weighted *R*-factor *wR* and goodness of fit *S* are based on *F*^2^, conventional *R*-factors *R* are based on *F*, with *F* set to zero for negative *F*^2^. The threshold expression of *F*^2^ > σ(*F*^2^) is used only for calculating *R*-factors(gt) *etc*. and is not relevant to the choice of reflections for refinement. *R*-factors based on *F*^2^ are statistically about twice as large as those based on *F*, and *R*- factors based on ALL data will be even larger.

### (II) Methyl 1-oxo-6-phenyl-2,3,4,12b-tetrahydro-1H,6H-chromeno[3,4-c]chromene-6a(7H)-carboxylate] Fractional atomic coordinates and isotropic or equivalent isotropic displacement parameters (Å^2^)

**Table:**

	*x*	*y*	*z*	*U*_iso_*/*U*_eq_	
C1	0.31613 (14)	0.23586 (8)	0.65866 (19)	0.0508 (4)	
H1	0.2975	0.2790	0.6860	0.061*	
C2	0.42353 (15)	0.20782 (10)	0.7195 (2)	0.0593 (4)	
H2	0.4769	0.2317	0.7894	0.071*	
C3	0.45178 (15)	0.14467 (10)	0.6769 (2)	0.0601 (4)	
H3	0.5257	0.1262	0.7147	0.072*	
C4	0.37039 (13)	0.10856 (8)	0.57786 (18)	0.0478 (4)	
H4	0.3898	0.0653	0.5521	0.057*	
C5	0.25975 (11)	0.13496 (7)	0.51513 (14)	0.0348 (3)	
C6	0.23532 (12)	0.19988 (7)	0.55629 (16)	0.0379 (3)	
C7	0.05795 (12)	0.20179 (7)	0.37858 (16)	0.0374 (3)	
H7A	0.0956	0.2085	0.2833	0.045*	
H7B	−0.0206	0.2229	0.3648	0.045*	
C8	0.04229 (11)	0.12785 (6)	0.40481 (14)	0.0311 (3)	
C9	0.16596 (11)	0.09252 (6)	0.41771 (14)	0.0316 (3)	
H9	0.1580	0.0510	0.4751	0.038*	
C10	0.20028 (12)	0.07429 (7)	0.25652 (15)	0.0376 (3)	
C11	0.12973 (14)	0.08917 (8)	0.12367 (16)	0.0436 (3)	
C12	−0.03692 (12)	0.09634 (7)	0.26444 (15)	0.0360 (3)	
H12	−0.0339	0.0480	0.2781	0.043*	
C13	0.29831 (14)	0.02678 (8)	0.2427 (2)	0.0486 (4)	
C14	0.33358 (18)	0.01282 (12)	0.0817 (2)	0.0733 (6)	
H14A	0.4203	0.0074	0.0892	0.088*	
H14B	0.2971	−0.0288	0.0442	0.088*	
C15	0.29703 (19)	0.06569 (12)	−0.0346 (2)	0.0751 (6)	
H15A	0.3138	0.0513	−0.1378	0.090*	
H15B	0.3437	0.1055	−0.0077	0.090*	
C16	0.16417 (17)	0.08128 (11)	−0.03816 (19)	0.0652 (5)	
H16A	0.1460	0.1219	−0.0968	0.078*	
H16B	0.1173	0.0457	−0.0911	0.078*	
C17	−0.16701 (13)	0.11648 (7)	0.23894 (16)	0.0418 (3)	
C18	−0.20700 (17)	0.16855 (9)	0.1421 (2)	0.0578 (4)	
H18	−0.1527	0.1939	0.0927	0.069*	
C19	−0.3299 (2)	0.18263 (11)	0.1194 (3)	0.0842 (7)	
H19	−0.3580	0.2171	0.0530	0.101*	
C20	−0.40961 (19)	0.14608 (13)	0.1939 (4)	0.0922 (8)	
H20	−0.4914	0.1560	0.1778	0.111*	
C21	−0.37047 (17)	0.09518 (12)	0.2915 (3)	0.0804 (6)	
H21	−0.4249	0.0709	0.3432	0.096*	
C22	−0.24989 (14)	0.08015 (9)	0.3127 (2)	0.0560 (4)	
H22	−0.2232	0.0450	0.3778	0.067*	
C23	−0.01905 (11)	0.11340 (6)	0.55155 (15)	0.0326 (3)	
C24	−0.16103 (16)	0.14882 (9)	0.7159 (2)	0.0607 (5)	
H24A	−0.2197	0.1152	0.6843	0.091*	
H24B	−0.2013	0.1887	0.7425	0.091*	
H24C	−0.1091	0.1336	0.8055	0.091*	
O1	0.12981 (9)	0.23212 (5)	0.50640 (12)	0.0447 (3)	
O2	0.01594 (10)	0.11195 (6)	0.12316 (11)	0.0471 (3)	
O3	0.34448 (11)	−0.00487 (7)	0.35498 (15)	0.0625 (3)	
O4	−0.00799 (10)	0.06241 (5)	0.62305 (12)	0.0489 (3)	
O5	−0.09014 (9)	0.16233 (5)	0.58848 (12)	0.0466 (3)	

### (II) Methyl 1-oxo-6-phenyl-2,3,4,12b-tetrahydro-1H,6H-chromeno[3,4-c]chromene-6a(7H)-carboxylate] Atomic displacement parameters (Å^2^)

**Table:**

	*U*^11^	*U*^22^	*U*^33^	*U*^12^	*U*^13^	*U*^23^
C1	0.0511 (9)	0.0473 (9)	0.0540 (9)	−0.0135 (7)	0.0058 (7)	−0.0108 (7)
C2	0.0479 (9)	0.0697 (12)	0.0575 (10)	−0.0193 (8)	−0.0071 (7)	−0.0041 (8)
C3	0.0403 (8)	0.0725 (12)	0.0642 (11)	−0.0054 (8)	−0.0084 (7)	0.0036 (9)
C4	0.0381 (7)	0.0530 (9)	0.0513 (9)	0.0026 (6)	−0.0001 (6)	−0.0004 (7)
C5	0.0338 (6)	0.0405 (7)	0.0303 (6)	−0.0017 (5)	0.0050 (5)	0.0011 (5)
C6	0.0361 (6)	0.0402 (7)	0.0383 (7)	−0.0044 (6)	0.0073 (5)	−0.0010 (5)
C7	0.0396 (7)	0.0326 (7)	0.0395 (7)	0.0011 (5)	0.0027 (5)	0.0026 (5)
C8	0.0317 (6)	0.0307 (6)	0.0305 (6)	0.0009 (5)	0.0025 (4)	0.0004 (5)
C9	0.0313 (6)	0.0316 (6)	0.0321 (6)	0.0024 (5)	0.0042 (5)	0.0006 (5)
C10	0.0394 (7)	0.0379 (7)	0.0368 (7)	−0.0015 (5)	0.0093 (5)	−0.0062 (5)
C11	0.0485 (8)	0.0467 (8)	0.0363 (7)	−0.0030 (6)	0.0082 (6)	−0.0066 (6)
C12	0.0384 (7)	0.0362 (7)	0.0323 (6)	0.0013 (5)	−0.0008 (5)	−0.0007 (5)
C13	0.0414 (8)	0.0502 (9)	0.0558 (9)	0.0002 (7)	0.0134 (7)	−0.0153 (7)
C14	0.0639 (11)	0.0924 (15)	0.0681 (12)	0.0066 (11)	0.0268 (9)	−0.0283 (11)
C15	0.0785 (13)	0.0995 (16)	0.0534 (11)	−0.0132 (12)	0.0334 (9)	−0.0195 (11)
C16	0.0760 (12)	0.0876 (14)	0.0339 (8)	−0.0055 (10)	0.0145 (8)	−0.0095 (8)
C17	0.0397 (7)	0.0412 (8)	0.0416 (7)	0.0045 (6)	−0.0079 (6)	−0.0072 (6)
C18	0.0592 (10)	0.0520 (10)	0.0576 (10)	0.0109 (8)	−0.0144 (8)	−0.0001 (7)
C19	0.0760 (14)	0.0629 (13)	0.1027 (17)	0.0275 (11)	−0.0389 (13)	−0.0101 (11)
C20	0.0431 (10)	0.0777 (16)	0.148 (2)	0.0128 (10)	−0.0234 (13)	−0.0324 (16)
C21	0.0398 (9)	0.0790 (15)	0.1203 (18)	−0.0055 (9)	0.0004 (11)	−0.0154 (13)
C22	0.0415 (8)	0.0573 (10)	0.0675 (11)	−0.0033 (7)	−0.0016 (7)	−0.0020 (8)
C23	0.0316 (6)	0.0338 (6)	0.0323 (6)	−0.0011 (5)	0.0027 (5)	−0.0029 (5)
C24	0.0606 (10)	0.0591 (10)	0.0691 (11)	0.0022 (8)	0.0366 (9)	−0.0021 (8)
O1	0.0432 (5)	0.0339 (5)	0.0560 (6)	−0.0005 (4)	0.0013 (4)	−0.0096 (4)
O2	0.0518 (6)	0.0586 (7)	0.0300 (5)	0.0053 (5)	0.0010 (4)	0.0005 (4)
O3	0.0557 (7)	0.0606 (8)	0.0711 (8)	0.0213 (6)	0.0069 (6)	−0.0092 (6)
O4	0.0620 (7)	0.0429 (6)	0.0442 (6)	0.0077 (5)	0.0161 (5)	0.0094 (4)
O5	0.0472 (6)	0.0415 (6)	0.0549 (6)	0.0055 (4)	0.0223 (5)	0.0020 (4)

### (II) Methyl 1-oxo-6-phenyl-2,3,4,12b-tetrahydro-1H,6H-chromeno[3,4-c]chromene-6a(7H)-carboxylate] Geometric parameters (Å, º)

**Table:**

C1—C2	1.375 (2)	C13—O3	1.220 (2)
C1—C6	1.391 (2)	C13—C14	1.506 (2)
C1—H1	0.9300	C14—C15	1.485 (3)
C2—C3	1.370 (3)	C14—H14A	0.9700
C2—H2	0.9300	C14—H14B	0.9700
C3—C4	1.380 (2)	C15—C16	1.514 (3)
C3—H3	0.9300	C15—H15A	0.9700
C4—C5	1.3965 (19)	C15—H15B	0.9700
C4—H4	0.9300	C16—H16A	0.9700
C5—C6	1.389 (2)	C16—H16B	0.9700
C5—C9	1.5265 (18)	C17—C18	1.381 (2)
C6—O1	1.3712 (17)	C17—C22	1.388 (2)
C7—O1	1.4224 (16)	C18—C19	1.394 (3)
C7—C8	1.5190 (18)	C18—H18	0.9300
C7—H7A	0.9700	C19—C20	1.368 (4)
C7—H7B	0.9700	C19—H19	0.9300
C8—C23	1.5280 (17)	C20—C21	1.365 (4)
C8—C9	1.5467 (17)	C20—H20	0.9300
C8—C12	1.5484 (18)	C21—C22	1.372 (2)
C9—C10	1.5216 (17)	C21—H21	0.9300
C9—H9	0.9800	C22—H22	0.9300
C10—C11	1.345 (2)	C23—O4	1.1957 (16)
C10—C13	1.469 (2)	C23—O5	1.3262 (15)
C11—O2	1.3508 (18)	C24—O5	1.4465 (17)
C11—C16	1.491 (2)	C24—H24A	0.9600
C12—O2	1.4418 (16)	C24—H24B	0.9600
C12—C17	1.5008 (19)	C24—H24C	0.9600
C12—H12	0.9800		
			
C2—C1—C6	120.18 (16)	C10—C13—C14	118.02 (16)
C2—C1—H1	119.9	C15—C14—C13	113.74 (16)
C6—C1—H1	119.9	C15—C14—H14A	108.8
C3—C2—C1	119.83 (15)	C13—C14—H14A	108.8
C3—C2—H2	120.1	C15—C14—H14B	108.8
C1—C2—H2	120.1	C13—C14—H14B	108.8
C2—C3—C4	119.87 (16)	H14A—C14—H14B	107.7
C2—C3—H3	120.1	C14—C15—C16	110.94 (16)
C4—C3—H3	120.1	C14—C15—H15A	109.5
C3—C4—C5	122.02 (16)	C16—C15—H15A	109.5
C3—C4—H4	119.0	C14—C15—H15B	109.5
C5—C4—H4	119.0	C16—C15—H15B	109.5
C6—C5—C4	116.79 (13)	H15A—C15—H15B	108.0
C6—C5—C9	121.60 (11)	C11—C16—C15	110.98 (15)
C4—C5—C9	121.41 (13)	C11—C16—H16A	109.4
O1—C6—C5	123.54 (12)	C15—C16—H16A	109.4
O1—C6—C1	115.13 (13)	C11—C16—H16B	109.4
C5—C6—C1	121.26 (14)	C15—C16—H16B	109.4
O1—C7—C8	111.79 (11)	H16A—C16—H16B	108.0
O1—C7—H7A	109.3	C18—C17—C22	119.22 (15)
C8—C7—H7A	109.3	C18—C17—C12	122.37 (15)
O1—C7—H7B	109.3	C22—C17—C12	118.39 (13)
C8—C7—H7B	109.3	C17—C18—C19	119.0 (2)
H7A—C7—H7B	107.9	C17—C18—H18	120.5
C7—C8—C23	112.26 (10)	C19—C18—H18	120.5
C7—C8—C9	110.17 (10)	C20—C19—C18	120.5 (2)
C23—C8—C9	109.45 (10)	C20—C19—H19	119.8
C7—C8—C12	110.59 (10)	C18—C19—H19	119.8
C23—C8—C12	107.06 (10)	C21—C20—C19	120.75 (19)
C9—C8—C12	107.14 (10)	C21—C20—H20	119.6
C10—C9—C5	113.94 (10)	C19—C20—H20	119.6
C10—C9—C8	111.11 (10)	C20—C21—C22	119.3 (2)
C5—C9—C8	109.51 (10)	C20—C21—H21	120.4
C10—C9—H9	107.3	C22—C21—H21	120.4
C5—C9—H9	107.3	C21—C22—C17	121.21 (19)
C8—C9—H9	107.3	C21—C22—H22	119.4
C11—C10—C13	116.61 (12)	C17—C22—H22	119.4
C11—C10—C9	122.25 (12)	O4—C23—O5	123.07 (12)
C13—C10—C9	119.78 (12)	O4—C23—C8	123.83 (11)
C10—C11—O2	122.67 (12)	O5—C23—C8	113.04 (11)
C10—C11—C16	125.32 (15)	O5—C24—H24A	109.5
O2—C11—C16	111.99 (13)	O5—C24—H24B	109.5
O2—C12—C17	107.44 (11)	H24A—C24—H24B	109.5
O2—C12—C8	108.24 (10)	O5—C24—H24C	109.5
C17—C12—C8	117.51 (11)	H24A—C24—H24C	109.5
O2—C12—H12	107.8	H24B—C24—H24C	109.5
C17—C12—H12	107.8	C6—O1—C7	115.35 (10)
C8—C12—H12	107.8	C11—O2—C12	113.41 (10)
O3—C13—C10	121.98 (14)	C23—O5—C24	115.74 (12)
O3—C13—C14	119.76 (15)		
			
C6—C1—C2—C3	1.1 (3)	C11—C10—C13—O3	−158.15 (16)
C1—C2—C3—C4	−2.5 (3)	C9—C10—C13—O3	8.8 (2)
C2—C3—C4—C5	1.7 (3)	C11—C10—C13—C14	16.1 (2)
C3—C4—C5—C6	0.4 (2)	C9—C10—C13—C14	−176.91 (14)
C3—C4—C5—C9	−174.44 (14)	O3—C13—C14—C15	−164.40 (17)
C4—C5—C6—O1	−178.79 (12)	C10—C13—C14—C15	21.2 (2)
C9—C5—C6—O1	−3.93 (19)	C13—C14—C15—C16	−52.5 (2)
C4—C5—C6—C1	−1.78 (19)	C10—C11—C16—C15	−10.0 (3)
C9—C5—C6—C1	173.08 (12)	O2—C11—C16—C15	171.53 (16)
C2—C1—C6—O1	178.29 (13)	C14—C15—C16—C11	46.8 (2)
C2—C1—C6—C5	1.0 (2)	O2—C12—C17—C18	−30.11 (18)
O1—C7—C8—C23	60.13 (14)	C8—C12—C17—C18	92.13 (16)
O1—C7—C8—C9	−62.12 (13)	O2—C12—C17—C22	148.25 (13)
O1—C7—C8—C12	179.62 (10)	C8—C12—C17—C22	−89.51 (17)
C6—C5—C9—C10	114.47 (13)	C22—C17—C18—C19	−0.9 (2)
C4—C5—C9—C10	−70.91 (16)	C12—C17—C18—C19	177.47 (16)
C6—C5—C9—C8	−10.65 (16)	C17—C18—C19—C20	1.0 (3)
C4—C5—C9—C8	163.97 (12)	C18—C19—C20—C21	−0.1 (4)
C7—C8—C9—C10	−85.59 (13)	C19—C20—C21—C22	−1.0 (4)
C23—C8—C9—C10	150.52 (11)	C20—C21—C22—C17	1.2 (3)
C12—C8—C9—C10	34.77 (14)	C18—C17—C22—C21	−0.2 (3)
C7—C8—C9—C5	41.15 (13)	C12—C17—C22—C21	−178.62 (16)
C23—C8—C9—C5	−82.74 (12)	C7—C8—C23—O4	−155.62 (13)
C12—C8—C9—C5	161.51 (10)	C9—C8—C23—O4	−32.96 (17)
C5—C9—C10—C11	−123.25 (14)	C12—C8—C23—O4	82.84 (15)
C8—C9—C10—C11	1.02 (18)	C7—C8—C23—O5	26.93 (15)
C5—C9—C10—C13	70.53 (16)	C9—C8—C23—O5	149.59 (11)
C8—C9—C10—C13	−165.20 (12)	C12—C8—C23—O5	−94.61 (12)
C13—C10—C11—O2	156.24 (14)	C5—C6—O1—C7	−15.15 (18)
C9—C10—C11—O2	−10.4 (2)	C1—C6—O1—C7	167.68 (12)
C13—C10—C11—C16	−22.1 (2)	C8—C7—O1—C6	48.13 (15)
C9—C10—C11—C16	171.31 (15)	C10—C11—O2—C12	−21.3 (2)
C7—C8—C12—O2	54.74 (13)	C16—C11—O2—C12	157.21 (13)
C23—C8—C12—O2	177.32 (10)	C17—C12—O2—C11	−172.70 (12)
C9—C8—C12—O2	−65.35 (13)	C8—C12—O2—C11	59.47 (14)
C7—C8—C12—C17	−67.09 (15)	O4—C23—O5—C24	−4.0 (2)
C23—C8—C12—C17	55.49 (15)	C8—C23—O5—C24	173.49 (12)
C9—C8—C12—C17	172.82 (11)		

### (II) Methyl 1-oxo-6-phenyl-2,3,4,12b-tetrahydro-1H,6H-chromeno[3,4-c]chromene-6a(7H)-carboxylate] Hydrogen-bond geometry (Å, º)

**Table:**

*D*—H···*A*	*D*—H	H···*A*	*D*···*A*	*D*—H···*A*
C4—H4···O3	0.93	2.22	2.973 (2)	138
C12—H12···O4^i^	0.98	2.41	3.3613 (18)	164

Symmetry code: (i) −*x*, −*y*, −*z*+1.

### (III) 6-(4-Ethylphenyl)-2,4-dimethyl-1,3-dioxo-2,3,4,12b-tetrahydro-1H,6H-chromeno[4',3':4,5]pyrano[2,3-d]pyrimidine-6a(7H)-carbonitrile] Crystal data

**Table:**

C_25_H_23_N_3_O_4_	*F*(000) = 904
*M**_r_* = 429.46	*D*_x_ = 1.345 Mg m^−^^3^
Monoclinic, *P*2_1_/*n*	Mo *K*α radiation, λ = 0.71073 Å
Hall symbol: -P 2yn	Cell parameters from 3715 reflections
*a* = 11.4471 (5) Å	θ = 2.1–25.0°
*b* = 11.2076 (4) Å	µ = 0.09 mm^−^^1^
*c* = 16.5407 (7) Å	*T* = 296 K
β = 91.990 (2)°	Block, colourless
*V* = 2120.80 (15) Å^3^	0.35 × 0.30 × 0.25 mm
*Z* = 4	

### (III) 6-(4-Ethylphenyl)-2,4-dimethyl-1,3-dioxo-2,3,4,12b-tetrahydro-1H,6H-chromeno[4',3':4,5]pyrano[2,3-d]pyrimidine-6a(7H)-carbonitrile] Data collection

**Table:**

Bruker Kappa APEXII CCD diffractometer	3715 independent reflections
Radiation source: fine-focus sealed tube	2814 reflections with *I* > 2σ(*I*)
Graphite monochromator	*R*_int_ = 0.030
ω & φ scans	θ_max_ = 25.0°, θ_min_ = 2.1°
Absorption correction: multi-scan (SADABS; Bruker, 2008)	*h* = −13→13
*T*_min_ = 0.968, *T*_max_ = 0.977	*k* = −10→13
18281 measured reflections	*l* = −19→19

### (III) 6-(4-Ethylphenyl)-2,4-dimethyl-1,3-dioxo-2,3,4,12b-tetrahydro-1H,6H-chromeno[4',3':4,5]pyrano[2,3-d]pyrimidine-6a(7H)-carbonitrile] Refinement

**Table:**

Refinement on *F*^2^	Primary atom site location: structure-invariant direct methods
Least-squares matrix: full	Secondary atom site location: difference Fourier map
*R*[*F*^2^ > 2σ(*F*^2^)] = 0.044	Hydrogen site location: inferred from neighbouring sites
*wR*(*F*^2^) = 0.126	H-atom parameters constrained
*S* = 1.03	*w* = 1/[σ^2^(*F*_o_^2^) + (0.0576*P*)^2^ + 0.8627*P*] where *P* = (*F*_o_^2^ + 2*F*_c_^2^)/3
3715 reflections	(Δ/σ)_max_ = 0.007
292 parameters	Δρ_max_ = 0.33 e Å^−^^3^
0 restraints	Δρ_min_ = −0.28 e Å^−^^3^

### (III) 6-(4-Ethylphenyl)-2,4-dimethyl-1,3-dioxo-2,3,4,12b-tetrahydro-1H,6H-chromeno[4',3':4,5]pyrano[2,3-d]pyrimidine-6a(7H)-carbonitrile] Special details

**Table:**

Geometry. All e.s.d.'s (except the e.s.d. in the dihedral angle between two l.s. planes) are estimated using the full covariance matrix. The cell e.s.d.'s are taken into account individually in the estimation of e.s.d.'s in distances, angles and torsion angles; correlations between e.s.d.'s in cell parameters are only used when they are defined by crystal symmetry. An approximate (isotropic) treatment of cell e.s.d.'s is used for estimating e.s.d.'s involving l.s. planes.
Refinement. Refinement of *F*^2^ against ALL reflections. The weighted *R*-factor *wR* and goodness of fit *S* are based on *F*^2^, conventional *R*-factors *R* are based on *F*, with *F* set to zero for negative *F*^2^. The threshold expression of *F*^2^ > σ(*F*^2^) is used only for calculating *R*-factors(gt) *etc*. and is not relevant to the choice of reflections for refinement. *R*-factors based on *F*^2^ are statistically about twice as large as those based on *F*, and *R*- factors based on ALL data will be even larger.

### (III) 6-(4-Ethylphenyl)-2,4-dimethyl-1,3-dioxo-2,3,4,12b-tetrahydro-1H,6H-chromeno[4',3':4,5]pyrano[2,3-d]pyrimidine-6a(7H)-carbonitrile] Fractional atomic coordinates and isotropic or equivalent isotropic displacement parameters (Å^2^)

**Table:**

	*x*	*y*	*z*	*U*_iso_*/*U*_eq_	
C1	0.1534 (2)	−0.1733 (2)	0.02179 (14)	0.0590 (6)	
H1	0.1589	−0.1965	−0.0319	0.071*	
C2	0.0885 (2)	−0.2402 (2)	0.07377 (15)	0.0609 (6)	
H2	0.0507	−0.3091	0.0555	0.073*	
C3	0.0802 (2)	−0.2042 (2)	0.15302 (15)	0.0565 (6)	
H3	0.0371	−0.2493	0.1886	0.068*	
C4	0.13542 (17)	−0.10173 (18)	0.17962 (13)	0.0465 (5)	
H4	0.1289	−0.0786	0.2333	0.056*	
C5	0.20076 (16)	−0.03175 (17)	0.12840 (11)	0.0396 (4)	
C6	0.21040 (18)	−0.07139 (18)	0.04949 (12)	0.0468 (5)	
C7	0.35811 (18)	0.06931 (19)	0.02593 (12)	0.0468 (5)	
H7A	0.3918	0.1143	−0.0176	0.056*	
H7B	0.4205	0.0255	0.0538	0.056*	
C8	0.30204 (16)	0.15565 (17)	0.08532 (11)	0.0373 (4)	
C9	0.25965 (16)	0.08300 (17)	0.15816 (11)	0.0372 (4)	
H9	0.3287	0.0606	0.1915	0.045*	
C10	0.18416 (15)	0.16192 (16)	0.20870 (11)	0.0359 (4)	
C11	0.13384 (15)	0.26105 (17)	0.17771 (10)	0.0353 (4)	
C12	0.19681 (16)	0.22106 (17)	0.04440 (11)	0.0374 (4)	
H12	0.1394	0.1608	0.0267	0.045*	
C13	0.17301 (16)	0.13736 (17)	0.29330 (11)	0.0391 (4)	
C14	0.04976 (16)	0.31618 (18)	0.30307 (12)	0.0409 (5)	
C15	0.01236 (19)	0.44176 (19)	0.18395 (13)	0.0522 (5)	
H15A	0.0704	0.4894	0.1586	0.078*	
H15B	−0.0251	0.4883	0.2243	0.078*	
H15C	−0.0449	0.4159	0.1439	0.078*	
C16	0.0767 (2)	0.1869 (2)	0.41949 (12)	0.0571 (6)	
H16A	0.1301	0.2304	0.4545	0.086*	
H16B	0.0866	0.1029	0.4287	0.086*	
H16C	−0.0021	0.2094	0.4306	0.086*	
C17	0.22490 (16)	0.29571 (17)	−0.02756 (11)	0.0378 (4)	
C18	0.2163 (2)	0.24542 (19)	−0.10328 (12)	0.0496 (5)	
H18	0.1908	0.1670	−0.1091	0.059*	
C19	0.2453 (2)	0.31004 (19)	−0.17082 (12)	0.0556 (6)	
H19	0.2390	0.2742	−0.2215	0.067*	
C20	0.28318 (19)	0.42640 (18)	−0.16474 (12)	0.0474 (5)	
C21	0.29008 (19)	0.47620 (18)	−0.08850 (12)	0.0490 (5)	
H21	0.3152	0.5548	−0.0828	0.059*	
C22	0.26093 (18)	0.41312 (17)	−0.02041 (12)	0.0443 (5)	
H22	0.2655	0.4495	0.0301	0.053*	
C23	0.3174 (3)	0.4970 (2)	−0.23790 (14)	0.0722 (7)	
H23A	0.2502	0.5435	−0.2564	0.087*	
H23B	0.3782	0.5528	−0.2210	0.087*	
C24	0.3588 (3)	0.4293 (3)	−0.30635 (16)	0.0839 (9)	
H24A	0.4161	0.3723	−0.2876	0.126*	
H24B	0.3932	0.4827	−0.3441	0.126*	
H24C	0.2942	0.3882	−0.3325	0.126*	
C25	0.39321 (18)	0.24049 (19)	0.11184 (12)	0.0438 (5)	
N1	0.10034 (14)	0.21462 (15)	0.33494 (9)	0.0413 (4)	
N2	0.06822 (13)	0.33754 (14)	0.22202 (9)	0.0395 (4)	
N3	0.46792 (18)	0.3023 (2)	0.13123 (13)	0.0693 (6)	
O1	0.27450 (14)	−0.01194 (13)	−0.00673 (8)	0.0555 (4)	
O2	0.14164 (11)	0.29896 (11)	0.10132 (7)	0.0415 (3)	
O3	0.22107 (12)	0.05425 (13)	0.32899 (8)	0.0499 (4)	
O4	−0.00801 (13)	0.38384 (13)	0.34285 (9)	0.0544 (4)	

### (III) 6-(4-Ethylphenyl)-2,4-dimethyl-1,3-dioxo-2,3,4,12b-tetrahydro-1H,6H-chromeno[4',3':4,5]pyrano[2,3-d]pyrimidine-6a(7H)-carbonitrile] Atomic displacement parameters (Å^2^)

**Table:**

	*U*^11^	*U*^22^	*U*^33^	*U*^12^	*U*^13^	*U*^23^
C1	0.0813 (16)	0.0442 (13)	0.0515 (13)	−0.0007 (12)	0.0011 (12)	−0.0045 (10)
C2	0.0706 (15)	0.0425 (13)	0.0691 (16)	−0.0078 (11)	−0.0053 (12)	0.0015 (12)
C3	0.0590 (13)	0.0445 (13)	0.0664 (15)	−0.0057 (10)	0.0076 (11)	0.0091 (11)
C4	0.0524 (12)	0.0405 (12)	0.0470 (11)	0.0016 (9)	0.0088 (9)	0.0058 (9)
C5	0.0433 (10)	0.0358 (11)	0.0400 (10)	0.0066 (8)	0.0066 (8)	0.0037 (8)
C6	0.0596 (12)	0.0367 (11)	0.0445 (11)	0.0040 (9)	0.0084 (10)	0.0039 (9)
C7	0.0562 (12)	0.0425 (12)	0.0427 (11)	0.0034 (10)	0.0172 (9)	0.0018 (9)
C8	0.0407 (10)	0.0363 (11)	0.0355 (10)	0.0006 (8)	0.0098 (8)	0.0019 (8)
C9	0.0405 (10)	0.0375 (11)	0.0341 (10)	0.0034 (8)	0.0081 (8)	0.0045 (8)
C10	0.0392 (9)	0.0361 (10)	0.0330 (9)	−0.0017 (8)	0.0097 (8)	0.0006 (8)
C11	0.0349 (9)	0.0385 (11)	0.0330 (9)	−0.0036 (8)	0.0081 (7)	0.0006 (8)
C12	0.0437 (10)	0.0361 (11)	0.0327 (9)	−0.0012 (8)	0.0086 (8)	0.0012 (8)
C13	0.0394 (10)	0.0402 (11)	0.0382 (10)	−0.0040 (9)	0.0080 (8)	0.0015 (9)
C14	0.0388 (10)	0.0436 (12)	0.0411 (11)	−0.0063 (9)	0.0114 (8)	−0.0045 (9)
C15	0.0535 (12)	0.0487 (13)	0.0550 (13)	0.0134 (10)	0.0117 (10)	0.0039 (10)
C16	0.0685 (14)	0.0674 (16)	0.0364 (11)	−0.0008 (12)	0.0178 (10)	0.0042 (10)
C17	0.0424 (10)	0.0369 (11)	0.0343 (10)	−0.0020 (8)	0.0051 (8)	0.0026 (8)
C18	0.0733 (14)	0.0363 (11)	0.0394 (11)	−0.0115 (10)	0.0070 (10)	−0.0011 (9)
C19	0.0886 (17)	0.0463 (13)	0.0323 (10)	−0.0067 (12)	0.0100 (10)	−0.0031 (9)
C20	0.0643 (13)	0.0396 (12)	0.0391 (11)	−0.0005 (10)	0.0109 (9)	0.0050 (9)
C21	0.0699 (14)	0.0332 (11)	0.0443 (11)	−0.0060 (10)	0.0062 (10)	0.0022 (9)
C22	0.0610 (12)	0.0363 (11)	0.0359 (10)	−0.0038 (9)	0.0050 (9)	−0.0010 (8)
C23	0.114 (2)	0.0560 (15)	0.0477 (13)	−0.0055 (14)	0.0223 (14)	0.0121 (12)
C24	0.116 (2)	0.0790 (19)	0.0596 (16)	0.0160 (17)	0.0368 (15)	0.0218 (14)
C25	0.0440 (11)	0.0488 (12)	0.0392 (10)	0.0005 (10)	0.0107 (9)	0.0037 (9)
N1	0.0469 (9)	0.0451 (10)	0.0326 (8)	−0.0020 (8)	0.0118 (7)	0.0003 (7)
N2	0.0408 (8)	0.0376 (9)	0.0409 (9)	0.0034 (7)	0.0110 (7)	0.0004 (7)
N3	0.0617 (12)	0.0812 (15)	0.0654 (13)	−0.0214 (12)	0.0054 (10)	−0.0054 (11)
O1	0.0826 (11)	0.0449 (9)	0.0402 (8)	−0.0055 (8)	0.0187 (7)	−0.0042 (7)
O2	0.0500 (8)	0.0403 (8)	0.0349 (7)	0.0083 (6)	0.0118 (6)	0.0060 (6)
O3	0.0603 (9)	0.0507 (9)	0.0390 (7)	0.0067 (7)	0.0071 (6)	0.0089 (7)
O4	0.0604 (9)	0.0524 (9)	0.0518 (9)	0.0056 (7)	0.0219 (7)	−0.0091 (7)

### (III) 6-(4-Ethylphenyl)-2,4-dimethyl-1,3-dioxo-2,3,4,12b-tetrahydro-1H,6H-chromeno[4',3':4,5]pyrano[2,3-d]pyrimidine-6a(7H)-carbonitrile] Geometric parameters (Å, º)

**Table:**

C1—C2	1.378 (3)	C14—O4	1.215 (2)
C1—C6	1.385 (3)	C14—N1	1.374 (3)
C1—H1	0.9300	C14—N2	1.385 (2)
C2—C3	1.378 (3)	C15—N2	1.463 (3)
C2—H2	0.9300	C15—H15A	0.9600
C3—C4	1.376 (3)	C15—H15B	0.9600
C3—H3	0.9300	C15—H15C	0.9600
C4—C5	1.392 (3)	C16—N1	1.467 (2)
C4—H4	0.9300	C16—H16A	0.9600
C5—C6	1.387 (3)	C16—H16B	0.9600
C5—C9	1.526 (3)	C16—H16C	0.9600
C6—O1	1.376 (2)	C17—C18	1.374 (3)
C7—O1	1.415 (2)	C17—C22	1.383 (3)
C7—C8	1.535 (3)	C18—C19	1.381 (3)
C7—H7A	0.9700	C18—H18	0.9300
C7—H7B	0.9700	C19—C20	1.377 (3)
C8—C25	1.468 (3)	C19—H19	0.9300
C8—C12	1.546 (3)	C20—C21	1.379 (3)
C8—C9	1.546 (2)	C20—C23	1.508 (3)
C9—C10	1.509 (2)	C21—C22	1.381 (3)
C9—H9	0.9800	C21—H21	0.9300
C10—C11	1.345 (3)	C22—H22	0.9300
C10—C13	1.436 (3)	C23—C24	1.456 (3)
C11—O2	1.339 (2)	C23—H23A	0.9700
C11—N2	1.369 (2)	C23—H23B	0.9700
C12—O2	1.445 (2)	C24—H24A	0.9600
C12—C17	1.499 (2)	C24—H24B	0.9600
C12—H12	0.9800	C24—H24C	0.9600
C13—O3	1.223 (2)	C25—N3	1.138 (3)
C13—N1	1.399 (2)		
			
C2—C1—C6	120.0 (2)	N1—C14—N2	115.99 (16)
C2—C1—H1	120.0	N2—C15—H15A	109.5
C6—C1—H1	120.0	N2—C15—H15B	109.5
C1—C2—C3	119.4 (2)	H15A—C15—H15B	109.5
C1—C2—H2	120.3	N2—C15—H15C	109.5
C3—C2—H2	120.3	H15A—C15—H15C	109.5
C4—C3—C2	120.1 (2)	H15B—C15—H15C	109.5
C4—C3—H3	119.9	N1—C16—H16A	109.5
C2—C3—H3	119.9	N1—C16—H16B	109.5
C3—C4—C5	121.8 (2)	H16A—C16—H16B	109.5
C3—C4—H4	119.1	N1—C16—H16C	109.5
C5—C4—H4	119.1	H16A—C16—H16C	109.5
C6—C5—C4	117.03 (18)	H16B—C16—H16C	109.5
C6—C5—C9	121.66 (17)	C18—C17—C22	118.68 (17)
C4—C5—C9	121.31 (17)	C18—C17—C12	118.97 (17)
O1—C6—C1	115.57 (19)	C22—C17—C12	122.33 (17)
O1—C6—C5	122.86 (18)	C17—C18—C19	120.71 (19)
C1—C6—C5	121.6 (2)	C17—C18—H18	119.6
O1—C7—C8	110.96 (16)	C19—C18—H18	119.6
O1—C7—H7A	109.4	C20—C19—C18	121.42 (19)
C8—C7—H7A	109.4	C20—C19—H19	119.3
O1—C7—H7B	109.4	C18—C19—H19	119.3
C8—C7—H7B	109.4	C19—C20—C21	117.26 (18)
H7A—C7—H7B	108.0	C19—C20—C23	121.85 (19)
C25—C8—C7	106.91 (15)	C21—C20—C23	120.9 (2)
C25—C8—C12	111.03 (16)	C20—C21—C22	122.08 (19)
C7—C8—C12	110.82 (15)	C20—C21—H21	119.0
C25—C8—C9	110.33 (15)	C22—C21—H21	119.0
C7—C8—C9	108.43 (16)	C21—C22—C17	119.83 (18)
C12—C8—C9	109.27 (14)	C21—C22—H22	120.1
C10—C9—C5	114.68 (15)	C17—C22—H22	120.1
C10—C9—C8	108.92 (15)	C24—C23—C20	116.8 (2)
C5—C9—C8	109.86 (15)	C24—C23—H23A	108.1
C10—C9—H9	107.7	C20—C23—H23A	108.1
C5—C9—H9	107.7	C24—C23—H23B	108.1
C8—C9—H9	107.7	C20—C23—H23B	108.1
C11—C10—C13	118.58 (17)	H23A—C23—H23B	107.3
C11—C10—C9	121.25 (16)	C23—C24—H24A	109.5
C13—C10—C9	119.96 (16)	C23—C24—H24B	109.5
O2—C11—C10	125.46 (16)	H24A—C24—H24B	109.5
O2—C11—N2	111.24 (16)	C23—C24—H24C	109.5
C10—C11—N2	123.29 (16)	H24A—C24—H24C	109.5
O2—C12—C17	106.90 (14)	H24B—C24—H24C	109.5
O2—C12—C8	110.65 (14)	N3—C25—C8	176.6 (2)
C17—C12—C8	115.27 (15)	C14—N1—C13	125.03 (16)
O2—C12—H12	107.9	C14—N1—C16	116.85 (16)
C17—C12—H12	107.9	C13—N1—C16	118.12 (17)
C8—C12—H12	107.9	C11—N2—C14	120.98 (16)
O3—C13—N1	119.93 (17)	C11—N2—C15	120.60 (16)
O3—C13—C10	124.23 (17)	C14—N2—C15	118.38 (16)
N1—C13—C10	115.83 (17)	C6—O1—C7	115.02 (15)
O4—C14—N1	122.65 (18)	C11—O2—C12	117.91 (14)
O4—C14—N2	121.36 (19)		
			
C6—C1—C2—C3	0.6 (4)	C9—C10—C13—N1	−179.40 (15)
C1—C2—C3—C4	0.6 (3)	O2—C12—C17—C18	−144.56 (18)
C2—C3—C4—C5	−0.1 (3)	C8—C12—C17—C18	92.0 (2)
C3—C4—C5—C6	−1.6 (3)	O2—C12—C17—C22	36.5 (2)
C3—C4—C5—C9	178.79 (18)	C8—C12—C17—C22	−87.0 (2)
C2—C1—C6—O1	178.9 (2)	C22—C17—C18—C19	1.2 (3)
C2—C1—C6—C5	−2.3 (3)	C12—C17—C18—C19	−177.8 (2)
C4—C5—C6—O1	−178.57 (18)	C17—C18—C19—C20	−0.2 (4)
C9—C5—C6—O1	1.1 (3)	C18—C19—C20—C21	−0.6 (3)
C4—C5—C6—C1	2.8 (3)	C18—C19—C20—C23	178.7 (2)
C9—C5—C6—C1	−177.61 (19)	C19—C20—C21—C22	0.2 (3)
O1—C7—C8—C25	−176.52 (15)	C23—C20—C21—C22	−179.1 (2)
O1—C7—C8—C12	−55.4 (2)	C20—C21—C22—C17	0.8 (3)
O1—C7—C8—C9	64.5 (2)	C18—C17—C22—C21	−1.5 (3)
C6—C5—C9—C10	135.93 (18)	C12—C17—C22—C21	177.45 (18)
C4—C5—C9—C10	−44.5 (2)	C19—C20—C23—C24	−26.5 (4)
C6—C5—C9—C8	12.9 (2)	C21—C20—C23—C24	152.7 (3)
C4—C5—C9—C8	−167.51 (17)	O4—C14—N1—C13	−176.16 (18)
C25—C8—C9—C10	74.10 (19)	N2—C14—N1—C13	3.9 (3)
C7—C8—C9—C10	−169.13 (15)	O4—C14—N1—C16	3.1 (3)
C12—C8—C9—C10	−48.2 (2)	N2—C14—N1—C16	−176.82 (17)
C25—C8—C9—C5	−159.52 (15)	O3—C13—N1—C14	174.31 (18)
C7—C8—C9—C5	−42.75 (19)	C10—C13—N1—C14	−6.6 (3)
C12—C8—C9—C5	78.14 (18)	O3—C13—N1—C16	−4.9 (3)
C5—C9—C10—C11	−103.3 (2)	C10—C13—N1—C16	174.15 (17)
C8—C9—C10—C11	20.3 (2)	O2—C11—N2—C14	−179.32 (15)
C5—C9—C10—C13	82.0 (2)	C10—C11—N2—C14	0.0 (3)
C8—C9—C10—C13	−154.41 (16)	O2—C11—N2—C15	2.9 (2)
C13—C10—C11—O2	176.38 (16)	C10—C11—N2—C15	−177.83 (18)
C9—C10—C11—O2	1.6 (3)	O4—C14—N2—C11	179.73 (17)
C13—C10—C11—N2	−2.8 (3)	N1—C14—N2—C11	−0.4 (3)
C9—C10—C11—N2	−177.56 (16)	O4—C14—N2—C15	−2.4 (3)
C25—C8—C12—O2	−63.45 (19)	N1—C14—N2—C15	177.48 (16)
C7—C8—C12—O2	177.89 (14)	C1—C6—O1—C7	−162.40 (19)
C9—C8—C12—O2	58.46 (19)	C5—C6—O1—C7	18.9 (3)
C25—C8—C12—C17	58.0 (2)	C8—C7—O1—C6	−51.8 (2)
C7—C8—C12—C17	−60.7 (2)	C10—C11—O2—C12	7.7 (3)
C9—C8—C12—C17	179.90 (15)	N2—C11—O2—C12	−173.07 (14)
C11—C10—C13—O3	−175.21 (18)	C17—C12—O2—C11	−164.19 (15)
C9—C10—C13—O3	−0.4 (3)	C8—C12—O2—C11	−37.9 (2)
C11—C10—C13—N1	5.8 (3)		

### (III) 6-(4-Ethylphenyl)-2,4-dimethyl-1,3-dioxo-2,3,4,12b-tetrahydro-1H,6H-chromeno[4',3':4,5]pyrano[2,3-d]pyrimidine-6a(7H)-carbonitrile] Hydrogen-bond geometry (Å, º)

Cg1 and Cg2 are the centroids of rings C14–C19 and C1–C6, respectively.

**Table:**

*D*—H···*A*	*D*—H	H···*A*	*D*···*A*	*D*—H···*A*
C4—H4···O3	0.93	2.39	3.155 (3)	139
C7—H7*B*···O4^i^	0.97	2.52	3.423 (3)	156
C15—H15*A*···O3^ii^	0.96	2.50	3.315 (3)	143
C16—H16*C*···*Cg*1^iii^	0.96	2.93	3.739 (2)	143
C24—H24*A*···*Cg*2^iv^	0.96	2.70	3.634 (3)	164

Symmetry codes: (i) −*x*+1/2, *y*−1/2, −*z*+1/2; (ii) −*x*+1/2, *y*+1/2, −*z*+1/2; (iii) *x*−3/2, −*y*−1/2, *z*−1/2; (iv) *x*−1/2, −*y*−1/2, *z*−3/2.

## References

[bb1] Andrade-Neto, V. F. de, Goulart, M. O. F., da Silva Filho, J. F., da Silva, M. J., Pinto, M. C. F. R., do, C. F. R., Pinto, A. V., Zalis, M. G., Carvalho, L. H. & Krettli, A. U. (2004). *Bioorg. Med. Chem. Lett.* **14**, 1145–1149. 10.1016/j.bmcl.2003.12.069 14980653

[bb16] Andrani, L. L. & Lapi, E. (1960). *Boll. Chim. Farm.* **99**, 583–586. 13759798

[bb2] Bakthadoss, M., Sivakumar, G. & Kannan, D. (2009). *Org. Lett.* **11**, 4466–4469. 10.1021/ol901228j 19775188

[bb3] Bhat, M. A., Siddiqui, N. & Khan, S. A. (2008). *Acta. Pol. Pharm.* **65**, 235–239. 18666431

[bb4] Bianchi, G. & Tava, A. (1987). *Agric. Biol. Chem.* **51**, 2001–2002.

[bb5] Bruker (2008). *APEX2*, *SAINT* and *SADABS*. Bruker AXS Inc., Madison, Wisconsin, USA.

[bb6] Cheng, J.-F., Ishikawa, A., Ono, Y., Arrhenius, T. & Nadzan, A. (2003). *Bioorg. Med. Chem. Lett.* **13**, 3647–3650. 10.1016/j.bmcl.2003.08.025 14552749

[bb9] Farrugia, L. J. (2012). *J. Appl. Cryst.* **45**, 849–854.

[bb10] Gourdeau, H., Leblond, L., Hamelin, B., Desputeau, C., Dong, K., Kianicka, I., Custeau, D., Boudreau, C., Geerts, L., Cai, S.-X., Drewe, J., Labrecque, D., Kasibhatla, S. & Tseng, B. (2004). *Mol. Cancer Ther.* **3**, 1375–1384. 15542776

[bb11] Groom, C. R. & Allen, F. H. (2014). *Angew. Chem. Int. Ed.* **53**, 662–671. 10.1002/anie.201306438 24382699

[bb12] Jain, N., Xu, J., Kanojia, R. M., Du, F., Jian-Zhong, G., Pacia, E., Lai, M.-T., Musto, A., Allan, G., Reuman, M., Li, X., Hahn, D. W., Cousineau, M., Peng, S., Ritchie, D., Russell, R., Lundeen, S. & Sui, Z. (2009). *J. Med. Chem.* **52**, 7544–7569. 10.1021/jm900146e 19366247

[bb13] Kanchanadevi, J., Anbalagan, G., Sivakumar, G., Bakthadoss, M. & Manivannan, V. (2011). *Acta Cryst.* E**67**, o1990. 10.1107/S160053681102678X PMC3213446 22091025

[bb14] Kathiravan, S. & Raghunathan, R. (2010). *Synlett*, **13**, 1927–1930.

[bb15] Khairy, A. M., Mohsen, M. A., Yahia, A. M., Basyouni, W. M. & Samir, Y. A. (2009). *WJC*, **4**, 161–170.

[bb17] Macrae, C. F., Bruno, I. J., Chisholm, J. A., Edgington, P. R., McCabe, P., Pidcock, E., Rodriguez-Monge, L., Taylor, R., van de Streek, J. & Wood, P. A. (2008). *J. Appl. Cryst.* **41**, 466–470.

[bb18] Mladenović, M., Mihailović, M., Bogojević, D., Matić, S., Nićiforović, N., Mihailović, V., Vuković, N., Sukdolak, S. & Solujić, S. (2011). *Int. J. Mol. Sci.* **12**(5), 2822–2841. 10.3390/ijms12052822 PMC3116159 21686153

[bb19] Mohr, S. J., Chirigos, M. A., Fuhrman, F. S. & Pryor, J. W. (1975). *Cancer Res.* **35**, 3750–3754. 1192431

[bb20] Moon, D.-O., Choi, Y. H., Kim, N.-D., Park, Y.-M. & Kim, G.-Y. (2007). *Int. Immunopharmacol.* **7**, 506–514. 10.1016/j.intimp.2006.12.006 17321474

[bb21] Ponnusamy, R., Sabari, V., Sivakumar, G., Bakthadoss, M. & Aravindhan, S. (2013). *Acta Cryst.* E**69**, o267–o268. 10.1107/S1600536813001244 PMC3569797 23424543

[bb7] Sangani, C. B., Shah, N. M., Patel, M. P. & Patel, R. G. (2012). *J. Serb. Chem. Soc.* **77**, 1165–1174.

[bb22] Sheldrick, G. M. (2008). *Acta Cryst.* A**64**, 112–122. 10.1107/S0108767307043930 18156677

[bb23] Smith, P. W., Sollis, S. L., Howes, P. D., Cherry, P. C., Starkey, I. D., Cobley, K. N., Weston, H., Scicinski, J., Merritt, A., Whittington, A., Wyatt, P., Taylor, N., Green, D., Bethell, R., Madar, S., Fenton, R. J., Morley, P. J., Pateman, T. & Beresford, A. (1998). *J. Med. Chem.* **41**, 787–797. 10.1021/jm970374b 9526555

[bb24] Spek, A. L. (2009). *Acta Cryst.* D**65**, 148–155. 10.1107/S090744490804362X PMC2631630 19171970

[bb26] Swaminathan, K., Sethusankar, K., Sivakumar, G. & Bakthadoss, M. (2011). *Acta Cryst.* E**67**, o2673. 10.1107/S1600536811037196 PMC3201225 22065629

[bb25] Thareja, S., Verma, A., Kalra, A., Gosain, S., Rewatkar, P. V. & Kokil, G. R. (2010). *Acta. Pol. Pharm.* **67**, 423–427. 20635539

[bb27] Tripathi, R. P., Tripathi, R., Bhaduri, A. P., Singh, S. N., Chatterjee, R. K. & Murthy, P. K. (2000). *Acta Trop.* **76**, 101–106. 10.1016/s0001-706x(00)00070-x 10936568

[bb28] Zhang, Y. L., Chen, B. Z., Zheng, K. Q., Xu, M. L., Lei, X. H. & Yaoxue, X. B. (1982). *Chem. Abstr.* **96**, 135383e.

